# Porcine γδ T cells express cytotoxic cell-associated markers and display killing activity but are not selectively cytotoxic against PRRSV- or swIAV-infected macrophages

**DOI:** 10.3389/fimmu.2024.1434011

**Published:** 2024-07-31

**Authors:** Leonie Bettin, Joseph Darbellay, Jill van Kessel, Neeraj Dhar, Volker Gerdts

**Affiliations:** ^1^ Vaccine and Infectious Disease Organization (VIDO), University of Saskatchewan, Saskatoon, SK, Canada; ^2^ Department of Veterinary Microbiology, Western College of Veterinary Medicine, University of Saskatchewan, Saskatoon, SK, Canada; ^3^ Department of Biochemistry, Microbiology, and Immunology, University of Saskatchewan, Saskatoon, SK, Canada; ^4^ School of Public Health, University of Saskatchewan, Saskatoon, SK, Canada

**Keywords:** Porcine γδ T cells, phenotype, cytotoxicity, PAMs, PRRSV, Influenza A Virus

## Abstract

**Background:**

Gamma-delta (γδ) T cells are a major immune cell subset in pigs. Approximately 50% of circulating T cells are γδ T cells in young pigs and up to 30% in adult sows. Despite this abundance, the functions of porcine γδ T cells are mostly unidentified. In humans and mice, activated γδ T cells exhibit broad innate cytotoxic activity against a wide variety of stressed, infected, and cancerous cells through death receptor/ligand-dependent and perforin/granzyme-dependent pathways. However, so far, it is unknown whether porcine γδ T cells have the ability to perform cytotoxic functions.

**Methods:**

In this study, we conducted a comprehensive phenotypic characterization of porcine γδ T cells isolated from blood, lung, and nasal mucosa. To further analyze the cytolytic potential of γδ T cells, *in vitro* cytotoxicity assays were performed using purified γδ T cells as effector cells and virus-exposed or mock-treated primary porcine alveolar macrophages as target cells.

**Results:**

Our results show that only CD2^+^ γδ T cells express cytotoxic markers (CD16, NKp46, perforin) with higher perforin and NKp46 expression in γδ T cells isolated from lung and nasal mucosa. Moreover, we found that γδ T cells can exhibit cytotoxic functions in a cell-cell contact and degranulation-dependent manner. However, porcine γδ T cells did not seem to specifically target Porcine Reproductive and Respiratory Syndrome Virus or swine Influenza A Virus-infected macrophages, which may be due to viral escape mechanisms.

**Conclusion:**

Porcine γδ T cells express cytotoxic markers and can exhibit cytotoxic activity in vitro. The specific mechanisms by which porcine γδ T cells recognize target cells are not fully understood but may involve the detection of cellular stress signals.

## 1 Introduction

Gamma-delta T cells are a subset of T cells expressing the γδ T cell receptor (TCR). They constitute a minor population of circulating T cells in humans, mice and dogs (~5%) but can make up more than 50% of the circulating T cells in pigs, cattle and chickens under physiological conditions (1–6). Gamma-delta T cells are the first T cell to develop in the embryonic thymus in many species, including mice, humans and pigs (7–9). The γδ T cells exiting the thymus during the embryonic development home to peripheral tissues such as the dermis, lungs, intestine and peripheral lymphoid organs (10). This homing behaviour and certain γδ T cell functions have been associated with the usage of specific TCR Vγ chains (mice) or Vδ chains (humans). For example, the Vγ9^+^Vδ2^+^ T cells subset in humans is predominantly found in the peripheral blood, can be activated by phosphoantigens, and produces TNFα and IFNγ upon activation (11).

A functional characterization based on TCR Vγ and Vδ chain usage has not been carried out for porcine γδ T cells, and hence other cell surface markers, in particular CD2 and CD8α, have been traditionally used to divide porcine γδ T cells into the following subsets: CD2^−^CD8α^−^, CD2^−^CD8α^dim^, CD2^+^CD8α^+^ and CD2^+^CD8α^−^. Although the functional relevance of this classification is still unclear, some functional differences between these subsets have been reported. For example, under IL-12, IL-18, IL-2, and ConA stimulation, mainly CD2^+^, not CD2^−^, γδ T cells produce IFNγ (12). Furthermore, we discovered that a co-stimulation with TLR7/8 ligand induces IFNγ production predominantly in CD2^+^ γδ T cells (1).

Gamma-delta T cell development and ligand recognition are complex and distinctly different from αβ T cells. A diverse array of γδTCR ligands and co-stimulatory signals has been discovered in humans and mice. Interestingly, these ligands are predominantly host-cell-derived molecules such as MHC-like, Ig-like, and B7 family-like proteins and phosphoantigens (13). This stands in stark contrast to the antigen recognition performed by αβ T cells, which is restricted to pathogen-derived peptides presented in MHC molecules. In terms of immune functions, human and murine γδ T cells exhibit a wide range of effector functions with innate and adaptive-like characteristics (14). One characteristic that has been explored extensively is γδ T cells’ ability to kill malignant, stressed or infected target cells, similar to cytotoxic T lymphocytes (CTL) and Natural Killer (NK) cells (15). Cytotoxic lymphocytes require a TCR-dependent activation to differentiate into effector cells and subsequently identify their target cells in an antigen and MHCI-restricted manner (16). In contrast, human and murine γδ T cells have been shown to use a broad spectrum of MHC-unrestricted mechanisms for target cell recognition. For instance, human Vγ9Vδ2 T cells lysed *Plasmodium falciparum*-infected red blood cells in a γδTCR, phosphoantigen and BTN3A1-dependent manner (17). Gamma-delta T cells can also express an array of activation receptors, traditionally known to be expressed by NK cells. These activating receptors are involved in the recognition of target cells by, for example, binding ligands that indicate cell stress (e.g. MICA, MICB, ULBP). Li et al. (18) and Qin et al. (19) showed that phosphoantigen-expanded human γδ T cells express the activating receptor NKG2D and kill Influenza A Virus (IAV) infected cells at a significantly higher rate than the mock controls. This cytolytic activity was at least partly dependent on NKG2D expression, as the blockade of this receptor reduced cytotoxicity by 30-70%. The molecular process of killing the target cell is comparable among all cytotoxic effector cells. The main mechanisms are the release of cytotoxic granules containing perforin and granzymes and the interaction between death receptors (on target cells) and death ligands (on effector cells). Both of these mechanisms have been reported for γδ T cell-mediated killing in humans and mice (19–24).

Whether porcine γδ T cells have similar cytotoxic functions and can thereby contribute to pathogen clearance or stress surveillance is unknown and has not been comprehensively explored. Interestingly, a comparison of transcriptomic profiles of circulating CD2^+^CD8α^+^ γδ T cells and CD2^+^CD8α^−^ γδ T cells revealed a greater expression of genes related to cytotoxic functions in CD2^+^CD8α^+^ cells [e.g. GNLY (granulysin), FCGR3A* (CD16), KLRK1 (NKG2D), GZMA (granzyme A), NKG7 (expressed in cytotoxic granules), GZMB (granzyme B)] (25). In accordance, a study done by Yang and Parkhouse (26) indicates that CD8α-expressing γδ T cells have cytotoxic effector functions. Porcine peripheral blood mononuclear cells (PBMCs) were selectively depleted of certain lymphocyte subsets, and CD3-redirected cytotoxicity was evaluated against a ^51^C-labeled cell line expressing CD32. The depletion of γδ T cells reduced but did not abrogate the killing. However, the depletion of all CD8α-expressing cells completely abolished killing, indicating that CD8^−^ γδ T cells are not involved in the observed cytotoxic response (26). Moreover, Olin and colleagues (27) described some cytotoxic activity of purified porcine γδ T cells against the K562 cell line; however, detailed information on mechanisms or specific γδ T cell subsets was not provided.

Therefore, in this study, we aimed to characterize the potential cytotoxic activity of porcine γδ T cells by phenotypical and functional analysis. The phenotypical analysis of tissue-associated (lung, nasal mucosa) and circulating γδ T cells revealed that only CD2^+^ γδ T cells express markers that can be associated with cytotoxic functions (CD16, NKp46, perforin). Furthermore, we utilized primary γδ T cells and autologous primary alveolar macrophages (PAMs) from healthy commercial pigs to establish an *in vitro* model system for the investigation of direct γδ T cell-mediated cytotoxicity. Swine Influenza A Virus (swIAV) and Porcine Reproductive and Respiratory Syndrome Virus (PRRSV) infected PAMs were included to study the ability of γδ T cells to participate in targeted and virus-specific cytolytic activity. Our results indicate that porcine γδ T cells exhibit cytotoxic functions in a cell-cell contact and degranulation-dependent manner but are either unable to specifically detect virus-infected cells or the viruses used in this study escape γδ T cell-mediated killing.

## 2 Materials and methods

### 2.1 Animals

The pigs used for this study were healthy commercial pigs (Dutch Landrace cross), 7 weeks (± 4 days) old and housed at the Prairie Swine Centre (Saskatchewan) since birth. Pigs were euthanized using lethal injection (pentobarbital). All experiments were conducted at the University of Saskatchewan following ethical regulations established by the Canadian Council on Animal Care and approved by the University of Saskatchewan Animal Care Committee (AUP# 20200112).

### 2.2 Cell isolation

After euthanasia, snouts were sawn off the skull in front of the eyes, lungs removed, and blood collected. Tissue samples were maintained in cold collection buffer (PBS with 2 mM EDTA, 100 U/ml Penicillin-Streptomycin (Thermo Fisher Scientific, Waltham, MA, USA)). A bronchoalveolar lavage (BAL) was performed on some of the lungs to collect PAMs. A BAL procedure was performed two times on the isolated lungs with 500 ml of PBS supplemented with 2 mM EDTA. BAL fluid was then filtered and washed twice. PBMCs were isolated by Ficoll-Paque™ Plus (Cytiva Life Sciences, Marlborough, MA, USA) density gradient centrifugation. The nasal mucosa was stripped from the underlying cartilage in the nasal cavity (septum and conchae). Both the nasal mucosa and lung tissue (5 x 2 cm) from the right cranial lobe were cut into small pieces and then subjected to enzymatic digestion in RPMI medium (RPMI 1640; Thermo Fisher Scientific) containing 20 mM HEPES, 100 U/ml Penicillin-Streptomycin, 90 μg/ml Gentamicin and 300 U/ml Collagenase I (Thermo Fisher Scientific) and 25 U/ml DNase I (Stemcell Technologies, Vancouver, BC, Canada). After a 1.5h digestion at 37°C, digested tissue was passed through a sieve and 40μm cell strainer (Corning Incorporated, NC, USA). After washing, cells were resuspended in complete RPMI medium and layered onto Ficoll-Paque™ Plus (Cytiva Life Sciences). Complete RPMI medium is RPMI 1640 supplemented with 10% fetal bovine serum (Sigma Aldrich, St. Louis, MO, USA), 0.5 mM β-Mercaptoethanol (Sigma Aldrich), 1% Antibiotic/Antimycotic, 1% HEPES, 1% MEM Non-Essential Amino Acids (all Thermo Fisher Scientific). After centrifugation, lymphocytes appeared at the interface of RPMI medium and Ficoll and were collected and washed once in complete RPMI. Subsequently, red blood cells were lysed with RBC lysis solution (Thermo Fisher Scientific) and washed twice. Cell numbers and viability were assessed by an automated cell counter using trypan blue staining (LUNA-II, Logos Biosystems, Annandale, VA, USA). All cells were cryopreserved in freezing media (90% fetal bovine serum: 10% dimethyl sulfoxide (Sigma–Aldrich) prior to their use.

### 2.3 Magnetic-activated cell sorting of γδ T cells

Thawed PBMCs were washed with RPMI complete and resuspended in MACS Buffer (PBS, 0.5% BSA, 2 mM EDTA). After an additional washing step, PBMCs were incubated with an antibody against the TCR-δ chain (PGBL22A; Kingfisher Biotech, Saint Paul, MN, USA) for 30 min at 1 µg/10^7^ cells. After washing twice, a 15-minute incubation with IgG_1_ microbeads (20 µl/10^7^cells; Miltenyi Biotec, Bergisch Gladbach, Germany) followed. Following the incubation with microbeads, γδ T cells were separated from other immune cells with two consecutive applied LS columns using a MidiMACS™ Separator following the manufacturer’s instructions (Miltenyi Biotec). Purity was assessed after every magnetic-activated cell sorting via flow cytometry and ranged between 97.8-99.5% (mean of 98.6%). After a resting period (72h) at 1x10^6^ cells/ml in complete RPMI at 37°C, the γδ T cells were washed, counted, and plated in a fresh round-bottom 96-well plate for functional assays.

### 2.4 Viruses

The wild-type IAV used in this study is swine influenza A/swine/Saskatchewan/18789/2002/H1N1 (Sk02). IAV was propagated on Madin-Darby canine kidney (MDCK) cells. PRRSV strain VR2332 (ATCC, Manassas, VA, USA) was used in the study and propagated on MARC-145 cells. When severe cytopathic effect was observed, about 5 days post-infection, the supernatant was collected by centrifugation to serve as virus stock. Virus stocks were stored at −80°C. One aliquot of each strain was thawed, and viral titers were determined by titration on MARC-145 cells for the PRRSV (VR2332) strain and on MDCK cells for swIAV H1N1 (Sk02). Titers were calculated as the TCID50/mL (28). All experiments were performed with the same batch of viruses.

### 2.5 PAM infection and cell trace violet staining

PAMs were thawed and plated at a density of 1x10^6^ cells/mL the day prior to infection. On the day of infection, PAMs were harvested and washed. After counting, they were placed in DMEM (serum-free), and the virus was added at a multiplicity of infection (MOI) of 0.5. Mock-infected cells received media containing no virus. For infection with swIAV, TPCK-treated trypsin (Sigma Aldrich) was added to the media at 1 μg/ml. The infected and mock-infected cells were placed at 37 °C for 2 hours, after which they were washed to remove unbound virions, followed by labeling with Cell Trace Violet (1 μM; Thermo Fisher) according to manufacturer instructions. Fresh DMEM supplemented with 10% fetal bovine serum (Sigma Aldrich), 1% Antibiotic/Antimycotic, 1% HEPES, 1% MEM Non-Essential Amino Acids (all Thermo Fisher Scientific) was then added to the cells, and they were replaced in a 37°C incubator. At 6 (swIAV) or 18 (PRRSV) hours post inoculation (hpi), cells were analyzed for intracellular Influenza A nucleoprotein and PRRSV-N-protein via flow cytometry (antibody details listed in **Table 1**) and plated for functional assays.

**Table 1:** Primary antibodies and secondary reagents used for flow cytometric analysis.

Antigen	Clone	Isotype	Fluoro- chrome	Labeling Strategy	Primary antibody source	Secondary antibody source
Analysis of infection rate						
**PRRSV-NP**	SR30	IgG1	FITC*	Directly conjugated	RTI, LLC	*-*
**IAV NP**	D67J	IgG2a	FITC*	Directly conjugated	Thermo Fisher	*-*
**Live/Dead**	–	–	Near IR	–	Invitrogen	*-*
Immunophenotyping of γδ T cells (cytotoxic markers)						
**TCR-γδ**	PGBL22A	IgG1	AF647**	Directly conjugated	Kingfisher	-
**CD3**	BB23-8E6-8C8	IgG1	PerCPCy5.5	Directly conjugated	BD Bioscience	-
**CD2**	MSA4	IgG2a	PE	Secondary antibody	Kingfisher	Southern Biotech
**CD8α**	PT81B	IgG2b	BV650	Secondary antibody	Kingfisher	BD Bioscience
**CD16**	G7	IgG1	FITC	Directly conjugated	Bio-Rad	–
**Nkp46**	VIV KM1	IgG1	BV421	Secondary antibody	Bio-Rad	Biolegend
**Live/Dead**	–	–	Aqua	–	Invitrogen	–
**Perforin**	δG9	IgG2b	PE-CF594	Directly conjugated	BD Bioscience	–
Analysis of degranulation						
**CD107a**	4E9/11	IgG1	FITC	Directly conjugated	Bio-Rad	–
**Live/Dead**	–	–	Aqua	–	Invitrogen	-
Analysis of perforin expression						
**CD2**	MSA4	IgG2a	AF647	Secondary antibody	Kingfisher	-
**CD8α- biotin**	76-2-11	IgG2a	SA-PECy7	Secondary antibody	Thermo Fisher	Southern Biotech
**Live/Dead**	–	–	Aqua	–	Invitrogen	-
**Perforin**	δG9	IgG2b	PE-CF594	Directly conjugated	BD Bioscience	

*Used in parallel samples.

**Directly labeled with Alexa Fluor™ 647 Antibody Labeling Kit (Thermo Fisher).

### 2.6 γδ T cell–PAM coculture

Purified and rested γδ T cells were co-cultured with autologous PAMs at 37°C, 5% CO2 for 4 h in 96-well plates (Ultra Low Attachment plate, round bottom, Corning) with or without a transwell system (Corning™ HTS Transwell; 0.4μm pore size) to assess γδ T cell degranulation and perforin production (effector-to-target ratio 1:3) or PAM lysis (effector-to-target cell ratios of 3:1, 6:1, 12:1, 25:1, 50:1). Degranulation was assessed by CD107a staining and flow cytometry. Briefly, FITC-conjugated anti-CD107a mAb (IgG1, clone 4E9/11, Bio-Rad; final concentration 4 μg/ml) was added to the microcultures at the start of the co-culture. After one hour of incubation, the protein transport inhibitors Brefeldin A (1 μg/ml; BD Bioscience, San Jose, CA, USA) and Monensin (2 μg/ml; BD Bioscience) were added to prevent intracellular degradation of internalized CD107a-antibody complexes. Cells were then incubated for a further 3h at 37°C in 5% CO2. Gamma-delta T cell degranulation was inhibited by treating cells for 1h before co-culture with 4 mM EGTA (Sigma Aldrich) and 2 mM EGTA during the co-culture.

### 2.7 PAM lysis assay

To measure PAM lysis, PAMs (infected or mock), labeled with 1 μM Cell Trace Violet, were cultured with autologous γδ T cells at indicated effector-to-target cell ratios. Once combined, the γδ T cells and PAMs were briefly spun down to bring the cells together and placed in the incubator for 4 hours at 37°C. Additional wells containing only PAMs or only γδ T cells were included for control purposes. PAMs cultured without γδ T cells were used to determine background cell death. After the incubation, cells were washed in PBS and stained with the Live/Dead™ Fixable Near-IR Dead Cell Stain Kit (Thermo Fisher Scientific) according to the manufacturer’s instructions. Following the Live/Dead staining, cells were fixed in 2% paraformaldehyde and acquired on the flow cytometer the next day. Percentage cell lysis was calculated by subtracting background lysis from the % of cell death observed in co-culture with γδ T cells.

### 2.8 Flow cytometry

For the immunophenotyping of γδ T cells isolated from blood, lung or nasal mucosa and for the analysis of perforin expression in γδ T cells after co-culture with PAMs, cells were incubated for 30 min at 4°C with the primary antibodies, then washed twice with Flow Cytometry Buffer (PBS with 2 mM EDTA, 1% BSA, 2 mM NaN3) before incubation with the matching secondary antibodies. Free binding sites of the secondary antibodies were blocked by whole mouse IgG (Jackson Immuno Research, West Grove, PA) prior to the staining with directly conjugated antibodies. After two washes, viability was assessed using the Live/Dead™ Fixable Near-IR Dead Cell Stain Kit (Thermo Fisher Scientific) according to the manufacturer’s instructions. After labeling cell surface markers, samples were fixed and permeabilized with FoxP3/Transcription Factor Staining Buffer Set (eBioscience, San Diego, CA, USA) according to the manufacturer’s instructions. Only directly conjugated antibodies were used for intracellular staining. Cells were incubated with the conjugated antibodies binding intracellular targets for 45 min at 4°C in the dark in 1x Permeabilization buffer. Technical information about the antibodies used is listed in **Table 1**. All antibodies were titrated prior to their use. At least 100,000 events (immunophenotyping) or 4,000 PAMs were collected on a Beckman Coulter CYTOFLEX S™ (laser configuration: V4-Y4-R3-B2). Data were analyzed using KALUZA analysis software 2.1 (Beckman Coulter) and FlowJo software, version 10.7.1, with gates based on the fluorescence minus one (FMO) controls. Cells were subjected to dead cell and doublet (FSC-A vs. FSC-H) discrimination and further gated, as shown in **Supplementary Figures 1** and **2**. Compensation was calculated after measurement of single-color stained beads with the Invitrogen™ AbC™ Total Antibody Compensation Bead Kit and the Invitrogen™ ArC™ Amine Reactive Compensation Bead Kit (Thermo Fisher Scientific) according to the manufacturer’s instructions.

### 2.9 Live cell imaging

The ability of γδ T cells to induce target cell death was assessed by monitoring the loss of plasma membrane integrity in PAMs using live cell imaging. Rested γδ T cells were stained with CFSE for cell tracking (1μM; Thermo Fisher) for 20 min at 37°C. Excess dye was removed by adding a 4-fold excess volume of RPMI containing 10% fetal bovine serum and harvesting the cells by centrifugation. Additionally, overnight rested PAMs were harvested and washed. Gamma-delta T cells and PAMs were plated in μ-Dish 35 mm at an effector to target ratio (E:T) of 5:1. After plating cells, To-Pro-3 (Thermo Fisher), a cell-impermeable dye used to label cells with compromised plasma membranes, was added directly to the wells to yield a final dye concentration of 1μM. The dish was imaged at 3-minute intervals on an inverted wide-field fluorescent microscope (Thunder Imaging system, Leica Microsystems) for a duration of 4h. The samples were maintained at 37°C and 5% CO2 in an environmental chamber (Okolab). In each experiment, about 50-60 independent positions were imaged using a 63X/1.4 NA oil immersion objective (Leica Microsystems) and images were captured on brightfield, green (475ex/535em) and red (635ex/642em) channels using a scientific CMOS K8 camera (Leica Microsystems).

### 2.10 RNA isolation and qPCR analysis

Total RNA was extracted from PAM immediately after thawing (pre-culture), or RNA extraction took place after thawed PAMs were rested and infected, as outlined in 2.5. SwIAV-infected PAMs and their corresponding mock controls were harvested at 6 hpi, whereas PRRSV-infected PAMs and their corresponding mock control cells were harvested at 18 hpi. Detailed RNA isolation and qPCR analysis are outlined in Bettin et al. (1). In brief, the RNA of PAMs was extracted using the RNeasy Micro Plus RNA Isolation kit (Qiagen, Hilden, Germany) according to manufacturer’s instructions. RNA was quantified by fluorometric quantification (Invitrogen™ Qubit™ RNA high sensitivity (HS) Kit, Thermo Fisher Scientific) and transcribed to cDNA using the Applied Biosystems™ High-Capacity cDNA Reverse Transcription kit (Thermo Fisher Scientific) according to the manufacturer’s instructions. Diluted cDNA was used in 20 μl reactions using KAPA SYBR PCR Master Mix (KAPA Biosystems, Wilmington, MA, USA). The qPCR was performed using a Step One Plus thermocycler (Applied Biosystems). The qPCR conditions were 95 °C for 20 seconds, followed by 40 cycles with denaturation at 95 °C for 15 seconds and an annealing temperature of 60°C for 30 seconds. A melting curve was included in each run. Primer amplification efficiency was calculated for every primer pair according to the equation: qPCR efficiency = (10^[−1/slope]^ − 1) × 100. Primer sequences and efficiency calculations can be found in **Supplementary Table 1**.

### 2.11 Statistical analysis

For the statistical analysis of data, GraphPad Prism 9.5.0 software was used. Before using parametric statistical tests, the required assumption of normality was tested with Shapiro-Wilk’s test. The distribution was considered normal when p ≥ 0.05. The data were then analyzed using one-way or two-way ANOVA, depending on the number of independent variables. In case of significant results, a Tukey or Sidak *post hoc* test was conducted for pairwise comparisons. Data obtained in transwell experiments were analyzed by area under the curve, followed by one-way ANOVA. The levels of significance were p ≤ 0.05 (∗), p ≤ 0.01 (∗∗) and p ≤ 0.001 (∗∗∗).

## 3 Results

### 3.1 Porcine γδ T cells express cytotoxic markers (NKp46, perforin, CD16), which are almost exclusively associated with a CD2^+^ phenotype

**Figure 1:**
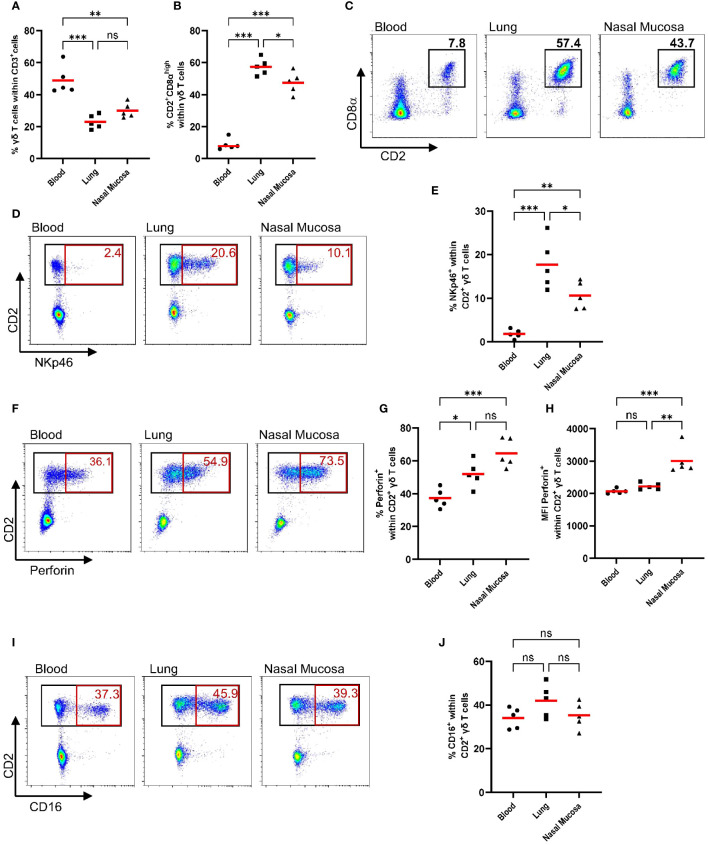
CD2^+^ γδ T cells express cytotoxic markers in circulation and in the respiratory tract (NKp46, Perforin, CD16). Previously cryopreserved lymphocytes from the blood, lung and nasal mucosa tissue were stained for flow cytometry immediately after thawing. During data analysis, dead cells and doublets (**Supplementary Figure 1**) were excluded and γδ T cell subsets (CD2^−^ and CD2^+^) were analyzed for their expression of cytotoxic markers (NKp46, Perforin, CD16). **(A)** The scatter diagram shows the percentage of γδ T cells within CD3^+^ cells in different tissues. Gamma-delta T cells (CD3^+^ TCR^+^) were then analyzed for their expression of CD8α and CD2 **(B)**, and representative plots from one pig are shown **(C)**. The expression of cytotoxic markers within CD2^+^ γδ T cells, including plots from representative animals, is depicted in D-J. **(D, E)** The frequency of NKp46^+^ cells within CD2^+^ γδ T cells for different tissues. **(F, G)** The frequency of perforin^+^ cells within CD2^+^ γδ T cells. **(H)** Median fluorescence intensity (MFI) of perforin expression within CD2^+^ γδ T cells from blood, lung or nasal mucosa. **(I, J)** The frequency of CD16^+^ cells within CD2^+^ γδ T cells. Each symbol represents data from one 7-week-old pig (n=5). Red bars indicate mean values, and the data were graphed in GraphPad Prism 9.5.0. The levels of significance were ns (= not significant), p ≤ 0.05 (∗), p ≤ 0.01 (∗∗) and p ≤ 0.001 (∗∗∗).

Human γδ T cells have been shown to exhibit cytotoxic activity against transformed, stressed or infected cells (29). However, as pointed out in two recent reviews (30, 31), it is not known if porcine γδ T cells display a similar cytotoxic activity. As a first step to evaluate the cytotoxic potential of porcine γδ T cells, we phenotypically characterized circulating γδ T cells and γδ T cells isolated from the respiratory tract for the expression of cytotoxic markers. Nasal mucosa and lung tissue were chosen to represent the upper and lower respiratory tract. Blood and tissue samples were obtained from healthy 7-week-old commercial pigs. The gating strategy shown in **Supplementary Figure 1** was used for all samples. Pigs are known for their high frequency of circulating γδ T cells, especially young pigs (1, 32). In line with previous studies, up to 60% of circulating T cells are γδ T cells (**Figure 1A**). As shown in **Figure 1A**, the percentage of T cells identified as γδ T cells is significantly lower in lung tissue and nasal mucosa than in circulation (mean of 49% in blood, 23% in lung tissue, 30.1% in nasal mucosa). Moreover, the distribution of CD2 and CD8α defined γδ T cell subsets is tissue-dependent. While the phenotype of CD2^+^CD8α^+^ γδ T cells accounted for only a small percentage of γδ T cells in the blood (mean of 9%), it was the main γδ T cell subset in lung tissue and nasal mucosa (**Figures 1B, C**). To further investigate the cytotoxic potential of γδ T cells, γδ T cells were analyzed for their expression of cytotoxic markers for which porcine-specific monoclonal antibodies have been developed, namely NKp46, perforin and CD16. NKp46 is one of the main receptors responsible for NK cell activation and has recently been shown to recognize externalized calreticulin, which translocates to the cell membrane during ER stress (33). Perforin is a pore-forming protein in the granules of NK cells or cytotoxic T cells and mediates the apoptosis of target cells (34). Furthermore, CD16, also known as FCγRIII, is a cell surface receptor that can bind to the Fc portion of IgG antibodies and is thereby involved in antibody-dependent cell-mediated cytotoxicity (35). As shown in **Figures 1D–J**, porcine γδ T cells express NKp46, perforin and CD16. Since CD2 is also a commonly used marker to classify porcine γδ T cells, its expression was analyzed in combination with NKp46, perforin and CD16. Notably, all of the measured cytotoxic markers were only expressed within CD2^+^ γδ T cells across all tissues tested (**Figures 1D, F, I** representative flow plots). Within the CD2^+^ γδ T cells, the majority of cytotoxic marker expressing cells were co-expressing CD8α at a high level. CD2^+^CD8α^−^ γδ T cells did not express NKp46, CD16 or perforin (**Supplementary Figures 3A, B**). In accordance with Mair et al. (36), NKp46 showed very low expression on circulating CD2^+^ γδ T cells (**Figures 1D, E**). However, almost 20% of CD2^+^ γδ T cells isolated from lung tissue expressed NKp46, which was also significantly higher expression than on CD2^+^ γδ T cells from the nasal mucosa (mean of 10.6%). Simultaneously, we analyzed NK cells (CD3^-^CD8α^+^) cytotoxic marker expression in the samples to provide a comparison to γδ T cells (**Supplementary Figure 4**). Compared to NK cells, the frequency of NKp46-positive cells within CD2^+^ γδ T cells was lower in every tissue tested. About 40% of NK cells from the lung are positive for NKp46, but the significant tissue-dependent differences observed for γδ T cells’ NKp46 expression were not seen for NK cells (**Supplementary Figure 4B**). While NK cells showed a high frequency of perforin expression in circulation (mean 79%) and a reduced expression in lung tissue (mean 60%; **Supplementary Figure 4C**), CD2^+^ γδ T cells showed the lowest frequency in circulation (mean 37%) and increased perforin expression in lung tissue and nasal mucosa. As shown in **Figure 1G**, the majority of CD2^+^ γδ T cells isolated from the nasal mucosa were positive for perforin (mean 65%). Moreover, the median fluorescence intensity (MFI) of perforin was consistently higher in CD2^+^ γδ T cells isolated from the nasal mucosa than from lung tissue or circulation (**Figure 1H**), which is similar to the pattern observed for NK cells (**Supplementary Figure 4B**). CD16 expression can be indicative of an antibody-dependent cytotoxic function or CD16-mediated phagocytosis and thereby identify a functional subset of γδ T cells. Recently, intestinal intraepithelial lymphocytes, especially CD2^+^CD8α^+^ γδ T cells, have been described as partly CD16^+^ (37, 38). In our study, CD16 showed a very distinct and consistent expression on CD2^+^ γδ T cells (mean 40%) across all anatomical locations tested (**Figures 1I, J**). In comparison to NK cells, the CD16 expression within CD2^+^ γδ T cells is lower. Almost all circulating NK cells are positive for CD16 (mean 93%), with a slightly lower expression on NK cells from lung tissue and nasal mucosa (**Supplementary Figure 4B**).

### 3.2 Porcine γδ T cells show cytotoxic activity, but specificity for virus-infected cells was not observed

The phenotypic characterization revealed that porcine γδ T cells, in particular CD2^+^ cells, express cytotoxic markers that could indicate a cytotoxic function of these cells. To further explore this potential cytotoxic function, we established an *in vitro* system to investigate γδ T cells as effector cells and virus- and mock-infected PAMs as target cells. PAMs are well-known primary target cells for PRRSV and have also been reported to be permissive to swIAV (39). As shown in **Supplementary Figure 5**, swIAV was able to infect PAMs and showed early and robust expression of intracellular viral nucleoprotein as early as 6 hpi. However, swIAV also induced a rapid cell death between 6-18 hpi, preventing us from using later time points for the γδ T cell cytotoxicity assays. Although PRRSV completes one infectious cycle in about 8h (40), the most robust and MOI-dependent expression of viral nucleoprotein was observed at 18 hpi with minimal changes in PAM viability (**Supplementary Figure 5**). Because of these virus-dependent differences in infection and replication dynamics in PAMs, different timelines had to be used for swIAV and PRRSV. SwIAV-infected macrophages and their corresponding mock control macrophages were used for functional assays between 5-9 hpi, while PRRSV-infected macrophages (and corresponding mock controls) were used between 17-21 hpi.

**Figure 2:**
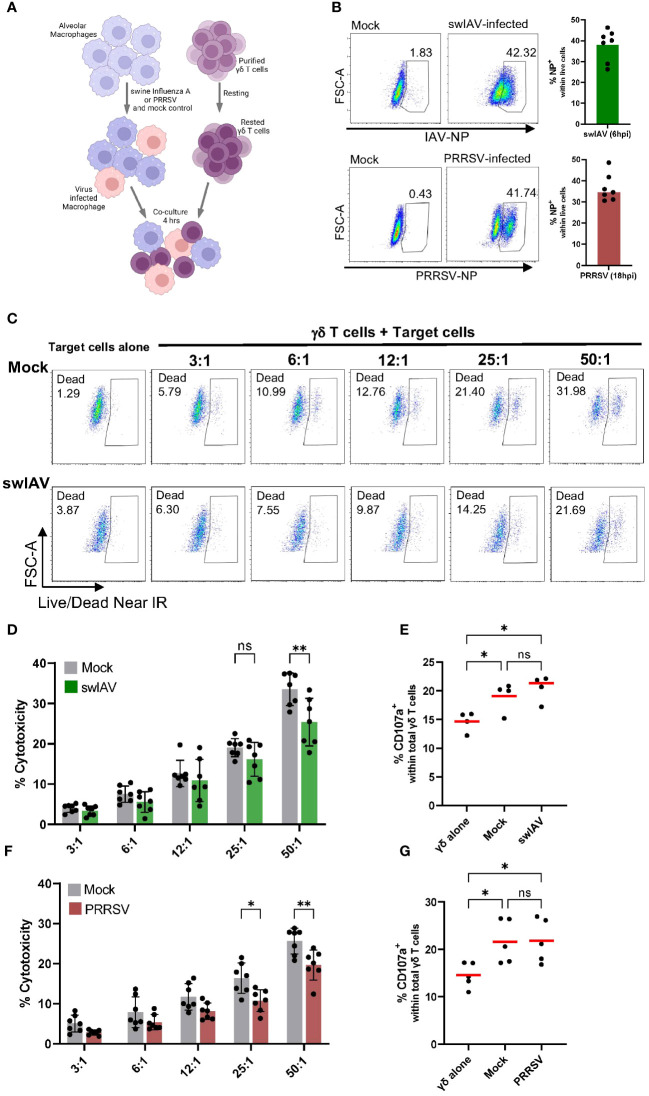
Porcine γδ T cells show cytotoxic activity against alveolar macrophages, but no specificity for virus-infected cells (swIAV or PRRSV) was observed. **(A)** Schematic illustrating the experimental design of γδ T cells killing assays. **(B)** Representative flow plots (left) and boxplots (right) showing the percentage of infected porcine alveolar macrophages (PAMs) as viral nucleoprotein+ cells following exposure with either swIAV (MOI = 0.5; 6 hpi) or PRRSV (MOI = 0.5; 18 hpi). A media (mock) control was included. Bar plots represent the mean of n=7. Each dot represents cells from an individual animal. **(C)** Representative flow plots showing the expression of viability dye in mock-treated or swIAV-exposed target cells without or with γδ T cells at varying target-to-effector ratios. The gating strategy is shown in **Supplementary Figure 2**. **(D, F)** Background-subtracted percentage of target cell death as measured by viability staining in uninfected cells (mock) and swIAV-exposed cells **(D)** or PRRSV-exposed cells **(F)** at different effector-to-target ratios. Background cell death for each experiment was calculated as the average of two wells of PAMs (mock or virus exposed) cultured without γδ T cells. Data are shown from n=7 individual 7-week-old pigs across 3 separate experiments. Mean values and standard deviation (SD) are shown. **(E, G)** Percentage of γδ T cells expressing CD107a upon culture with no targets, mock-treated targets or swIAV-exposed **(E)** or PRRSV-exposed **(G)** target cells. Each symbol represents data from one 7-week-old pig (n= 5). Red bars indicate mean values, and the data were graphed in GraphPad Prism 9.5.0. The levels of significance were ns (= not significant), p ≤ 0.05 (∗) and p ≤ 0.01 (∗∗).

To understand how exposure to macrophages (mock or virus infected) impacts γδ T cell phenotype and function, we added γδ T cells that had been positively isolated and rested to infected or mock-treated PAMs (**Figure 2A**). We then assessed the ability of these γδ T cells to lyse virus-exposed or mock-treated autologous target cells (PAMs). PAMs were infected with either swIAV (H1N1) or PRRSV (VR2332) at an MOI of 0.5, which resulted in an infection rate of about 35% after 6 hpi for swIAV and 18 hpi for PRRSV measured by intracellular staining for viral nucleoprotein (**Figure 2B**). Thus, the virus-infected PAMs are a mixture of infected PAMs and bystander cells and will be referred to as virus-exposed PAMs to reflect this composition.

As shown in **Figure 2C** for one representative animal, the co-culture with γδ T cells induced significant target cell death in both mock and swIAV-exposed PAMs. The increase in PAM cell death with increasing effector-to-target cell ratio was consistent for all seven animals tested (**Figure 2D**). Although a trend towards a decreased cytotoxic activity of γδ T cells when co-cultured with swIAV-exposed target cells was observed for almost all effector-to-target ratios, the most robust and statistically significant decrease was seen at the highest (50:1) ratio. Gamma-delta T cells co-cultured with PAMs also degranulated as assessed by the CD107a externalization (**Figure 2E**). The gating for CD107a expression is shown in **Supplementary Figure 6**. A significant difference in terms of γδ T cell degranulation between the co-culture with mock or swIAV-exposed PAMs was not present. Similar to swIAV, γδ T cells showed a slightly decreased cytotoxicity towards PRRSV-exposed PAMs, as shown in **Figure 2F**, in particular at effector-to-target ratios 25:1 and 50:1. However, γδ T cells degranulated at the same rate when co-cultured with mock or PRRSV-exposed PAMs (**Figure 2G**).

### 3.3 Gamma-delta T cells express perforin in response to short-term co-culture with PAMs

**Figure 3:**
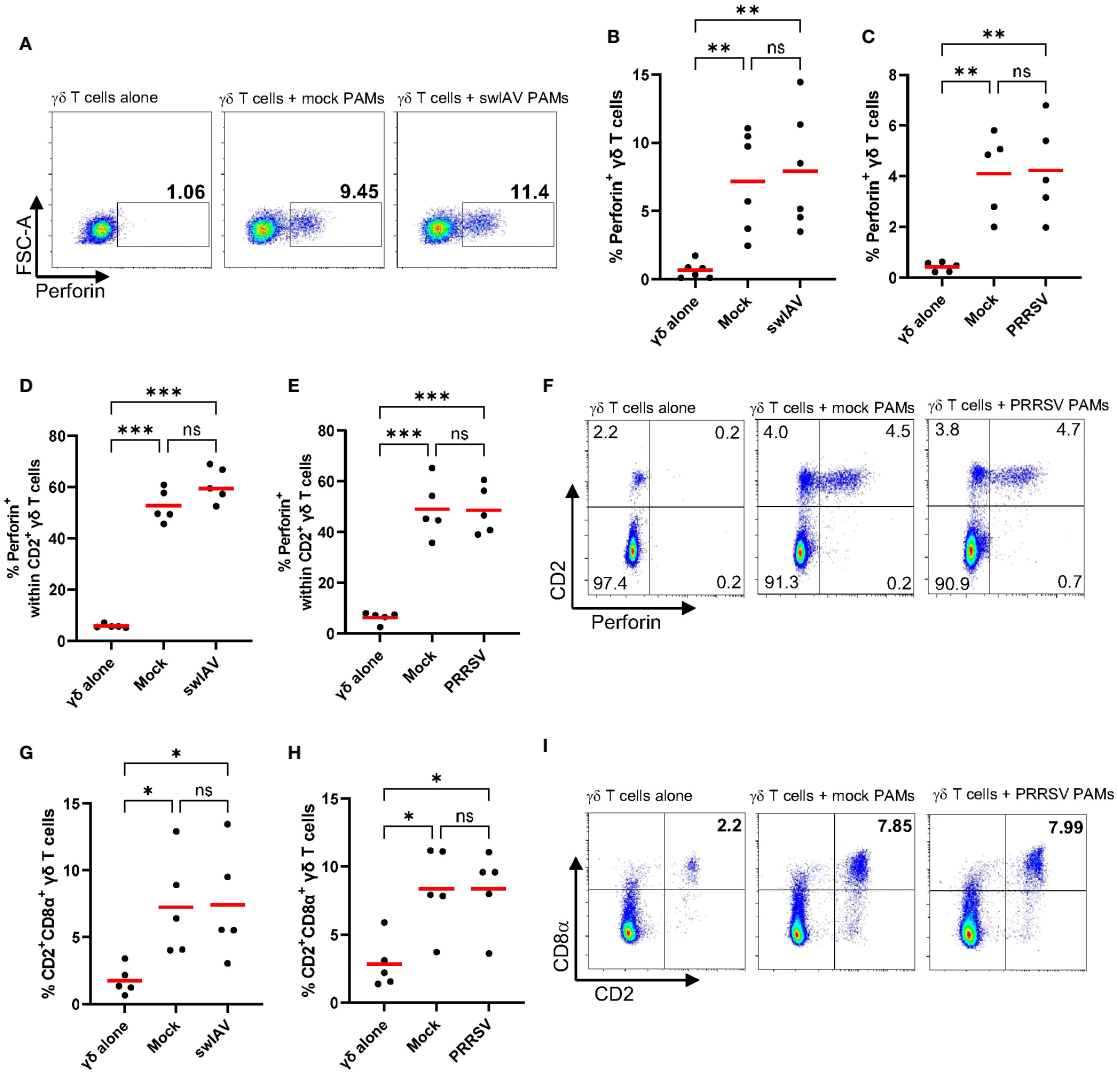
Frequency and phenotype of perforin expressing γδ T cells after short-term co-culture with PAMs. **(A)** Representative flow plots showing the percentage of perforin expressing γδ T cells within total γδ T cells after culturing them alone or after 4h of co-culture with mock-treated PAMs or swIAV-exposed PAMs. **(B, C)** Scatter plots showing the percentage of perforin^+^ γδ T cells within total γδ T cells in two experiments, including swIAV-exposed PAMs **(B)** or PRRSV-exposed PAMs **(C)** and their respective mock control. **(D, E)** Scatter plots showing the frequency of perforin^+^ cells within CD2^+^ γδ T cells when cultured alone or in co-culture with swIAV-exposed PAMs **(D)** or PRRSV-exposed PAMs **(E)**. **(F)** Representative flow plots showing γδ T cells and their CD2 and perforin expression under different culture conditions as indicated in the plots. Numbers located in quadrants indicate the percentage of cells for one particular phenotype. **(G, H)** Scatter plots showing the frequency of CD2^+^CD8α^+^ γδ T cells cultured alone or cultured with swIAV-exposed PAMs **(G)** or PRRSV-exposed PAMs **(H)**. **(I)** Representative flow plots showing γδ T cells and their CD2 and CD8α expression under different culture conditions as indicated in the plots. Each symbol represents data from one 7-week-old pig (n=5-6 per experiment). Red bars indicate mean values, and the data were graphed in GraphPad Prism 9.5.0. The levels of significance were ns (= not significant), p ≤ 0.05 (∗), p ≤ 0.01 (∗∗) and p ≤ 0.001 (∗∗∗).

We observed an increase in CD107a expression on γδ T cells after short-term (4h) co-culture with mock or virus-exposed PAMs, indicating that γδ T cells degranulate (**Figures 2E, G**). Degranulation is a cellular process that releases cytotoxic or other molecules from intracellular granules. Most commonly, perforin and granzymes are released during the process of degranulation. Thus, we aimed to analyze perforin expression within γδ T cells and coupled it with an analysis of γδ T cell subsets defined by CD2 and CD8α expression. As before, experiments were conducted with swIAV-exposed PAMs or PRRSV-exposed PAMs and their respective mock control. Rested γδ T cells (before co-culture) did not express perforin (mean 0.8%) and showed a low frequency of the CD2^+^CD8α^+^ phenotype (mean 2.3%) (**Supplementary Figure 7**). While γδ T cells cultured alone stayed negative for intracellular perforin, γδ T cells co-cultured with mock or virus-exposed PAMs increased perforin expression significantly, as shown for one representative pig in **Figure 3A**. **Figures 3B, C** show the expression of perforin within total γδ T cells for six individual pigs, and although some individual variation exists, the increase of perforin expression is evident for all pigs tested. A difference between mock and swIAV- or PRRSV-exposed PAMs was not detected in terms of γδ T cell perforin expression. The *ex vivo* staining for perforin (**Figure 1**) revealed that perforin is almost exclusively expressed by CD2^+^ γδ T cells, which also applies to this *in vitro* induction of perforin synthesis. Perforin is expressed at a high rate within CD2^+^ γδ T cells (up to 70%) after co-culture with PAMs (**Figures 3D, E**) and as shown for one representative animal, CD2^-^ γδ T cells do not express perforin (**Figure 3F**). The perforin expressing cells are not only positive for CD2 but also for CD8α (**Supplementary Figure 8**). In addition to the perforin synthesis by CD2^+^ γδ T cells, the frequency of CD2^+^CD8α^+^ γδ T cells within total γδ T cells increases significantly over the course of the short-term co-culture with mock or virus-exposed PAMs compared to γδ T cells cultured alone (**Figures 3G–I**).

**Figure 4:**
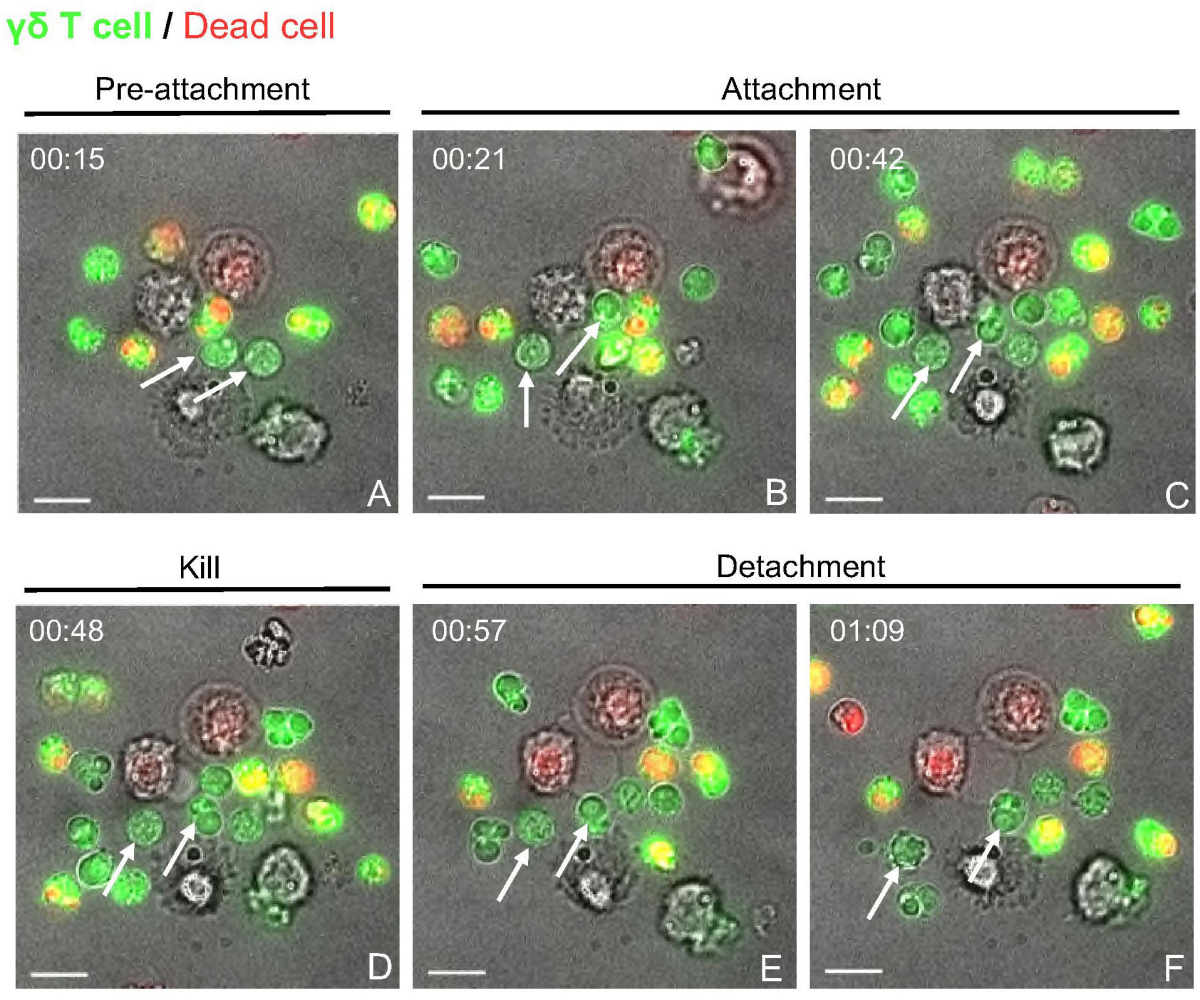
Live cell imaging of γδ T cell-PAM co-culture indicates cell-cell contact dependent cytotoxicity events. Time-lapse imaging was carried out over 4 h for γδ T cells cultured with untreated, but rested PAMs at an E:T ratio of 5:1 in μ-Dish 35 mm (ibidi). **(A)** Unconjugated γδ T cells (CFSE stained (green); white arrows). **(B, C)** Two γδ T cells attach to a macrophage (unstained). **(D)** depicts the To-pro-3 entry (red) into the macrophage, indicating killing. **(E, F)** shows the detachment of the γδ T cells from the dead target cell. Scale bar= 10 μm. Time is shown as hh:mm.

### 3.4 Gamma-delta T cell-mediated cytotoxicity depends on cell-cell contact and degranulation

**Figure 5:**
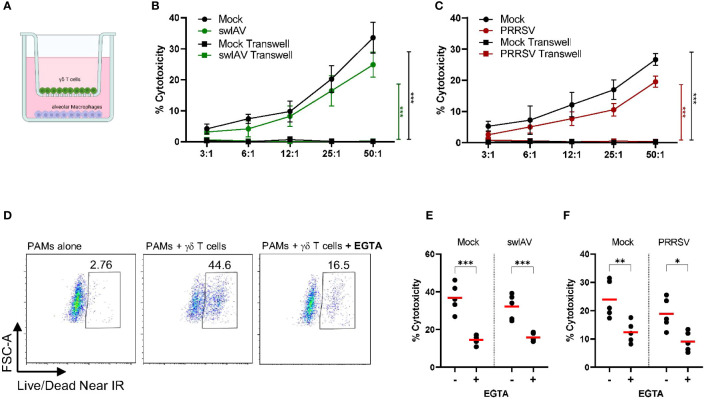
Porcine γδ T cell-mediated killing is cell-cell contact and extracellular Ca2^+^ dependent. **(A)** Schematic illustrating the transwell assay setup. Purified and rested γδ T cells and PAMs were cocultured for 4h in transwell plates with γδ T cells on top and macrophages on bottom. **(B, C)** shows the background-subtracted percentage of target cell death (PAMs) when cultured together with γδ T cells or separated by a transwell membrane at different effector: target ratios (n=3). PAMs were either exposed to swIAV **(B)** or PRRSV **(C)** before co-culture. Background cell death for each experiment was calculated as the average of two wells of PAMs (mock or virus exposed) cultured without γδ T cells. **(D)** Representative flow plots for one individual pig showing the expression of viability dye in mock-treated PAMs without or with γδ T cells and in the absence or presence of EGTA (E:T= 30:1). Gamma-delta T cells were added to swIAV-exposed **(E)** or PRRSV-exposed **(F)** or respective mock-treated PAMs in the absence or presence of EGTA. PAM lysis (cytotoxicity) was measured by flow cytometry at an E:T= 30:1 (n=5). Red bars indicate mean values, and the data were graphed in GraphPad Prism 9.5.0. The levels of significance were p ≤ 0.05 (∗), p ≤ 0.01 (∗∗) and p ≤ 0.001 (∗∗∗).

The flow cytometry-based cytotoxicity assays with γδ T cells and PAMs revealed a potential cytotoxic activity of porcine γδ T cells (**Figures 2** and **3**). The molecular process behind the killing of a target cell is comparable among all cytotoxic effector cells. It typically involves two main mechanisms: the release of cytotoxic granules (perforin, granzyme) or the binding of death ligands, expressed on effector cells, to death receptors, expressed on target cells. In both cases, the effector cell binds to the target cell, forming the so-called immunological synapse. In order to investigate if the observed γδ T cell mediated killing depends on similar mechanisms, the γδ T cell-PAM interactions were further studied at a single-cell level using live-cell imaging. **Figure 4** shows a representative killing event. In this time-lapse, a γδ T cell mediated killing event was captured involving the steps pre-attachment, attachment (conjugation), killing and detachment in a time span of ~ 1h. The entire time lapse in pictures is shown in **Supplementary Figure 9** and a video sequence has been added to the **Supplementary Material**. This visualization indicated that γδ T cell mediated cytotoxicity is cell contact-dependent and potentially resembles killing mechanisms by CTLs or NK cells. However, this qualitative data with cells from one individual pig was not conclusive enough to determine cell-cell contact dependency. Thus, flow cytometry-based cytotoxicity assays were repeated in combination with a transwell system, where a membrane separated the γδ T cells and PAMs. Hence, cell-cell contact was prevented, but the exchange of soluble factors was not (**Figure 5A**). Under these conditions, no killing of PAMs by γδ T cells occurred (**Figures 5B, C**). As shown before in **Figure 2**, a slight decrease in γδ T cell-mediated killing was observed in co-culture with swIAV or PRRSV-exposed PAMs compared to their respective mock control, especially at an E:T ratio of 50:1 (mean of 33.6% vs 24.8% and mean of 26.6% vs 19.5%, respectively).

Since we observed increased and potentially *de novo* production of perforin by γδ T cells after 4h of co-culture with virus-exposed or mock-treated PAMs and an increase of the degranulation marker CD107a, we hypothesized that γδ T cells can release cytotoxic granules, which in turn induce target cell killing. The degranulation process and the subsequent pore-forming function of perforin highly depend on extracellular Ca^2+^ (41, 42). Thus, a calcium chelator (EGTA) can be used to reduce the available Ca^2+^ in the media, thereby preventing degranulation events. The background cell death of PAMs (without γδ T cells) was unchanged in the presence of EGTA (**Supplementary Figure 10**). However, the PAM lysis observed in PAM-γδ T cell co-culture was strongly inhibited in the presence of EGTA for both mock-treated or swIAV- or PRRSV-exposed PAMs (**Figures 5D–F**). About 10-15% residual cytotoxicity remains present under EGTA treatment.

### 3.5 Mock and virus-exposed PAMs show an increased transcript expression of cell stress- or activation-induced ligands

**Figure 6:**
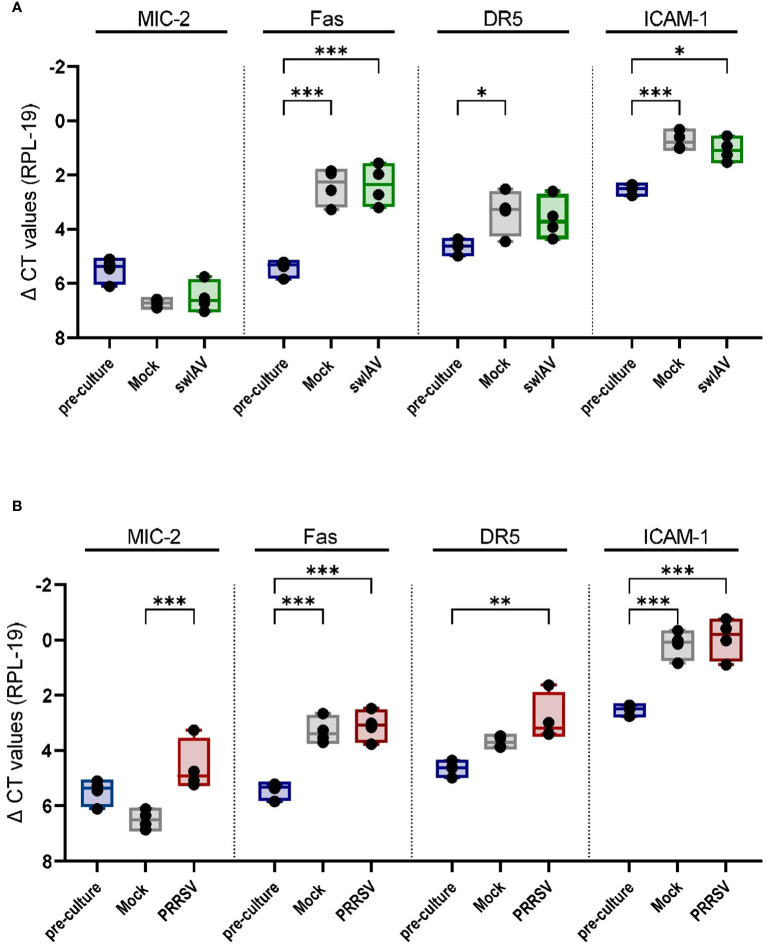
Mock treatment and viral exposure of PAMs increase the expression of Fas, DR5, and ICAM-1 compared to pre-culture macrophages. Boxplots show the delta CT values of MIC-2, Fas, DR5 and ICAM-1 in mock and **(A)** swIAV- or **(B)** PRRSV-exposed PAMs as measured by qPCR. Each point represents one individual pig and the mean of two qPCR technical replicates (n=4). The levels of significance were p ≤ 0.05 (∗), p ≤ 0.01 (∗∗) and p ≤ 0.001 (∗∗∗).

Interestingly, we observed a cytotoxic response of porcine γδ T cells against mock-treated PAMs. We hypothesize that this may be due to the handling of the cells and culture period, which could induce cell stress or activation and thereby trigger the recognition by cytotoxic effector cells like γδ T cells. To test this hypothesis, we analyzed the transcript expression of some potentially stress-induced ligands in PAMs pre-culture (harvested, cryopreserved and thawed) and mock-treated or infected PAMs (harvested, cryopreserved, thawed, rested, infected/mock-treated and cultured (~24h for swIAV and ~36h for PRRSV). MIC-2 is an MHC class I chain-related (MIC) protein whose function is not well characterized in pigs. However, in humans, MIC proteins (MICA/MICB) have been identified as a ligand of the activating receptor NKG2D and are upregulated in response to cellular stress (43). DR5 (Death Receptor 5; TNF receptor superfamily member 10b) and Fas are death receptors inducing cell death upon binding to their respective ligand (TRAIL and FasL). Both are constitutively expressed in most tissues and cell types but seem to be upregulated upon cellular activation or stress (44–46). ICAM-1 is an adhesion molecule crucial for the formation of the immunological synapse and is upregulated in response to various inflammatory stimuli (e.g. hypoxia, IFNγ, LPS) (47). As shown in **Figure 6**, all of the ligands listed above were expressed in non-cultured PAMs (pre-culture), especially ICAM-1. Nevertheless, the culture and infection/mock treatment significantly increased the expression of Fas and ICAM-1 transcript regardless of the viral infection (**Figures 6A, B**). DR5 transcript showed a slight increase under mock conditions (**Figure 6A**) or in PRRSV-exposed cells compared to non-cultured PAMs (**Figure 6B**). Interestingly, MIC2 expression showed an overall downward trend except for PRRSV-exposed PAMs.

## 4 Discussion

Human and murine γδ T cells display cytotoxic functions against a wide range of target cells. However, the contributions of porcine γδ T cells to the clearance of infected cells and stress surveillance have not been characterized. Thus, we aimed to analyze the cytotoxic potential of porcine γδ T cells. The phenotypical characterization of tissue-associated and circulating γδ T cells showed that cytotoxic markers (NKp46, perforin and CD16) are only expressed by CD2^+^ γδ T cells. NKp46 is an activating receptor, which is expressed by all human NK cells (48) and differentially expressed by porcine NK cells (NKp46^bright^, NKp46^+^ and NKp46^−^ subsets) (49, 50). To validate our NKp46 staining, porcine NK cells were analyzed in addition to γδ T cells and the above pattern was corroborated for all tissues analyzed. In our study, circulating γδ T cells showed minimal expression of NKp46, but up to 25% of CD2^+^ γδ T cells in lung tissue were NKp46^+^. The NKp46 expression by porcine T cells was also investigated by Mair and colleagues (36), and they determined that CD3^+^NKp46^+^ cells exist and a minor subset of those belongs to the γδ T cell population in blood. Moreover, Mair and colleagues described increased frequencies of CD3^+^NKp46^+^ cells in the lung, as well as the expression of NKp46 by CD2^+^ γδ T cells. Interestingly, transcript of other NK-associated receptors, like NKp30 or NKG2D, was found in CD3^+^NKp46^+^ cells at an NK-like level, which might indicate that NKp46^+^ γδ T cells express more activating receptors than investigated in our study. In line with the NK-like phenotype of some CD2^+^ γδ T cells, perforin expression was exclusively found in CD2^+^ γδ T cells. Rodríguez-Gómez et al. (51) and Stas et al. (52) also found that perforin expression by porcine γδ T cells is associated with a CD2^+^CD8α^+^CD27^−^ phenotype in blood, spleen and lung tissue and a frequent co-expression of T-bet. They speculated that this γδ T cell phenotype (CD2^+^CD8α^+^CD27^−^) could be indicative of terminally differentiated γδ T cells with effector functions, which seems to be supported by our findings that cytotoxic markers are only expressed by CD2^+^ γδ T cells. Interestingly, the highest perforin expression (in frequency and MFI) within CD2^+^ γδ T cells was found in γδ T cells isolated from the nasal mucosa. Little is known about γδ T cells or other immune cells in the nasal mucosa, but the nasal mucosa is constantly exposed to pollutants, pathogens and has a resident microflora. Thus, immune cells in the nasal region are exposed to a broad range of environmental stimuli and likely have a crucial role in early immune responses. The high perforin expression in nasal CD2^+^ γδ T cells could translate into an increased cytotoxic activity of nasal mucosa-associated γδ T cells. However, we are currently missing the functional studies supporting this. Notably, *in vivo* nasal CD8α^+^ γδ T cells showed a marked increase in perforin and T-bet expression 4 days after inoculation with swIAV, whereas lung and BAL γδ T cells only slightly increased perforin expression supporting the idea that nasal γδ T cells are part of an early anti-viral immune response (53). Like NKp46 and perforin, CD16 is exclusively expressed by a subset of CD2^+^ γδ T cells. CD16, also known as FCγRIII, binds the Fc portion of IgGs and is, therefore, a target cell recognition mechanism and indispensable for antibody-dependent cellular cytotoxicity (ADCC). CD16 on human and murine γδ T cells have been shown to have the same function as CD16 on NK cells, namely killing target cells upon binding of CD16 to the Fc portion of antibodies bound to a target cell (54, 55). A similar mechanism likely applies to porcine γδ T cells, but those studies have not been conducted thus far. Overall, it remains to be seen whether the increased perforin expression in nasal γδ T cells or the higher frequency of NKp46^+^ γδ T cells in lung tissue is indicative of an organ-specific modulation and translates into functional differences. Nevertheless, this phenotypic analysis indicated that cytotoxic responses by γδ T cells are possible and most likely mediated by CD2^+^CD8α^+^ γδ T cells. In order to confirm cytotoxic functions, we established an *in vitro* assay with γδ T cells as effector cells and PAMs (mock-treated or swIAV/PRRSV-exposed) as target cells. Curiously, we found that γδ T cells can kill autologous PAMs. Consistent with our findings, Cao et al. (56) noticed a high rate of PAM lysis mediated by porcine NK cells. They speculated that this may stem from stress stimuli during the *in vitro* cultivation (~24h), rendering PAMs susceptible to NK cell-mediated killing. Human NK cells have also been shown to lyse autologous monocyte-derived macrophages, which can be greatly increased by activating the macrophages with high doses of LPS, which results in an increased expression of stress ligands (e.g. MICA/MICB and ULBP3) (57). Moreover, IL-12 activated NK cells were able to kill autologous M0 and M2 macrophages in a mainly NKp46 and DNAM-1 dependent manner (58). Tramonti et al. (59) aimed to determine whether activated chemokine-producing macrophages are targets for murine γδ T cells. Thus, they isolated peritoneal macrophages from *Listeria monocytogenes* infected mice, which are known for their chemokine production, and co-cultured them with γδ T cells (Vγ1). The Vγ1+ T cells efficiently killed these macrophages using the Fas–FasL pathway. Similarly, *in vitro* expanded human Vδ2+ T cells have the ability to kill zoledronic acid treated, autologous monocyte-derived macrophages (21). Thus, NK cell or γδ T cell mediated killing towards autologous cells can be activated by a variety of external signals and is dependent on multiple recognition and killing pathways. The harvesting of PAMs, freeze/thaw and the culture period without host-tissue derived factors in our experiments are likely to have induced cellular stress or unintended activation, which could have triggered the γδ T cell mediated cytotoxicity observed. Although we used cell culture media with low levels of endotoxins (<0.01 EU/ml) to culture PAMs, we can not completely exclude that endotoxins were introduced through the addition of media supplements. Hence, endotoxins could be another mechanism by which PAMs were unintentionally stressed or activated. The increased expression of Fas, DR5 and ICAM-1 transcript in mock and virus-exposed macrophages supports this claim, as all of these receptors have been shown to be connected to cellular stress and activation (45, 46, 60, 61). Moreover, we noted less cytotoxicity from γδ T cells towards PAMs that were cryopreserved but not pre-cultured. At an E:T ratio of 50:1, we observed a mean cytotoxicity of 13.7% against non-cultured PAMs, compared to approximately 25-35% against mock-treated PAMs (**Figures 2D, F**, **Supplementary Figure 11**), which demonstrates the influence PAM culture conditions can have.

Notably, we did not observe a difference in Fas, DR5 or ICAM-1 transcript levels between mock and virus-exposed macrophages. The viral infection either does not influence the transcript expression of these receptors or the virus-induced cell stress has been masked by the overall cell stress the PAMs experienced in culture. Moreover, it should be noted that transcript abundance may not correlate with the corresponding protein expression. Thus, virus-exposed PAMs might differ from their mock controls in the expression of Fas, DR5, or ICAM-1 at the protein level but not at the transcript level, highlighting the need for future studies on the protein expression of stress ligands and their potential recognition by porcine γδ T cells.

Interestingly, the infection of PAMs with swIAV or PRRSV reduced the γδ T cell mediated killing by about ~10%. This 10% reduction in target cell lysis was also reported for PRRSV-infected PAMs co-cultured with NK cells. In the same study, NK cells killed pseudorabies virus-infected PAMs slightly more than mock control PAMs, indicating that the decreased lysis of PRRSV-infected PAMs is a PRRSV-specific mechanism (56). In contrast to PRRSV, which is known for its replication in macrophages, IAV infection of macrophages is less common but possible and can be either abortive or productive in a strain and macrophage subtype-specific manner (62–64). We observed that swIAV (H1N1) infected PAMs, but an early and marked cell death between 6-18 hpi was noted (**Supplementary Figure 5**). Similarly, Chang et al. (39) found that PAMs are susceptible to infection with influenza viruses, including swine H1N1, but acute and early apoptosis was only evident for avian influenza virus-infected PAMs, not swine H1N1 infected PAMs. Although the same influenza subtypes were used by Chang et al. (39) and us, different strains were utilized, which could account for the differences described. Due to the rapid cell death induced by the H1N1 strain utilized in our study, it is possible that some swIAV-infected PAMs entered the early phases of apoptosis during the 4h cytotoxicity assay. PAMs in early apoptosis might not be a target for γδ T cells, but due to the still intact cell membrane, they were counted as “alive” in our assay, skewing the results towards a slightly reduced lysis rate for swIAV-exposed PAMs. The PRRSV-induced cell death is slower and less drastic in the time frame the cytotoxicity assay took place (**Supplementary Figure 5**), making it less likely to be the only explanation for the reduced killing of PRRSV-exposed PAMs by γδ T cells. Notably, various viruses have developed strategies to escape the killing by cytotoxic effector cells. Especially IAV has been reported to evade NK cell mediated cytotoxicity through various mechanisms (65). For example, IAV (H1N1, H3N2) infection of DU145 cells results in an MHCI redistribution, which then allows for better recognition by NK cell inhibitory receptors and consequently inhibits NK cell cytotoxicity (66). The potential strategies adopted by PRRSV have not been studied, and underlying mechanisms that could lead to reduced cytotoxicity by effector cells are still unknown (67). Since the ligands and exact mechanisms through which porcine γδ T cells recognize target cells are unknown, it is difficult to test whether those are altered by swIAV or PRRSV infection.

Nevertheless, our data suggests that porcine γδ T cells have cytotoxic activity, which also seems to correlate with a *de novo* perforin expression upon contact with PAMs. The exact mechanism behind this induced expression of perforin remains unknown. However, the recognition of PAMPs/DAMPs or cytokines derived from PAMs could be a possible explanation. Rested γδ T cells (before co-culture) do not express perforin; however, upon co-culture with PAMs, perforin expression can be detected. This would indicate that a *de novo* perforin production occurs immediately following the γδ T cell activation upon contact with PAMs. In accordance, Makedonas et al. (68) showed that human CD8 T cells are capable of *de novo* perforin production following target cell recognition in as little as 1h. This newly synthesized perforin appeared in the Golgi and was not restricted to the granule compartment but still accumulated at the immunological synapse and took part in the target cell killing, as inhibiting protein synthesis in CD8 T cells significantly diminished the killing activity.

In accordance with our *ex vivo* phenotypic analysis, only CD2^+^CD8α^+^ γδ T cells showed perforin expression upon co-culture with PAMs. These findings align with previous studies that found greater expression of genes related to cytotoxic functions in CD2^+^CD8α^+^ γδ T cells (compared to CD2^+^CD8α^−^ γδ T cells) and indicated some cytotoxic functions for CD8α-expressing γδ T cells (25, 26). The hypothesis that only CD2^+^CD8α^+^ γδ T cells are responsible for the cytotoxic response observed is in agreement with the high effector-to-target ratios needed to see substantial killing of PAMs (50:1). The CD2^+^CD8α^+^ γδ T cells are only a minor subset of circulating γδ T cells in young pigs (~5-15%) and showed a frequency of ~8% in the co-cultures with PAMs. Thus, a high effector-to-target ratio is needed to increase the absolute number of these γδ T cells in culture. Subset-specific cytotoxicity assays will need to be conducted in the future to confirm this assumption.

The lysis of PAMs required direct contact between PAMs and γδ T cells since killing was abrogated entirely when γδ T cells and PAMs were separated by a membrane. Moreover, Ca^2+^-dependent mechanisms, likely γδ T cell degranulation, played a significant role as PAM lysis was reduced in the presence of EGTA, a calcium-chelating agent. Perforin-mediated cytotoxicity strictly depends on extracellular calcium, while death receptor/ligand interactions are less calcium-dependent (69). Since the presence of EGTA did not completely abrogate the γδ T cell-mediated cell death, it is possible that other cytotoxicity mechanisms like the Fas/FasL or DR5/TRAIL interaction take place, which would be in line with the increase in Fas and a slight increase in DR5 transcript expression by PAMs. However, we currently lack reagents to evaluate the FasL or TRAIL protein expression on porcine γδ T cells and selectively evaluate functions by blocking studies.

Collectively, our data shows that porcine γδ T cells express cytotoxic markers and can exhibit cytotoxic activity in a cell-cell contact and degranulation-dependent manner. Yet, the specific receptor-ligand interactions enabling porcine γδ T cells to recognize target cells are not fully understood, although they may entail the recognition of cellular stress.

## Data availability statement

The raw data supporting the conclusions of this article will be made available by the authors, without undue reservation.

## Ethics statement

The animal study was approved by University Animal Care Committee of the University of Saskatchewan. The study was conducted in accordance with the local legislation and institutional requirements.

## Author contributions

LB: Conceptualization, Data curation, Formal analysis, Investigation, Methodology, Validation, Visualization, Writing – original draft, Writing – review & editing. JD: Formal analysis, Methodology, Writing – original draft, Writing – review & editing. Jv: Methodology, Writing – original draft, Writing – review & editing. ND: Formal Analysis, Methodology, Writing – original draft, Writing – review & editing. VG: Conceptualization, Funding acquisition, Project administration, Supervision, Writing – original draft, Writing – review & editing.
