# Supplementary material for: Porcine γδ T cells express cytotoxic cell-associated markers and display killing activity but are not selectively cytotoxic against PRRSV- or swIAV-infected macrophages
Source: Front Immunol. 2024 Jul 31;15:1434011. doi: 10.3389/fimmu.2024.1434011 (PMC11321972; doi:10.3389/fimmu.2024.1434011)

Supplementary Material

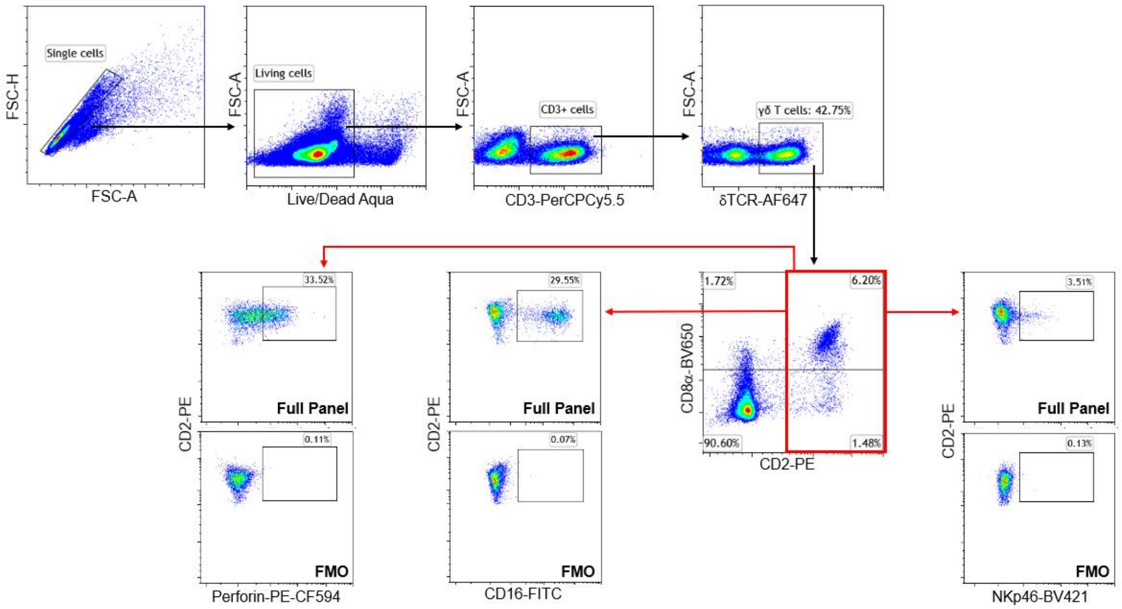

Supplementary Fig. 1. Schematic representation of the gating strategy for the analysis of cytotoxic markers expressed by γδ T cells. A gate was established around singlets before drawing a gate surrounding intact cells. After this doublet and dead cell exclusion, T cells were identified (CD3+) and the percentage of γδ T cells within T cells analyzed. Gamma-delta T cells were then further gated and analyzed for the expression of CD2 and CD8α, followed by the expression of NKp46, perforin or CD16 within $CD2^+$ γδ T cells. The Fluorescence minus one (FMO) control is shown for the NKp46, perforin and CD16 staining.

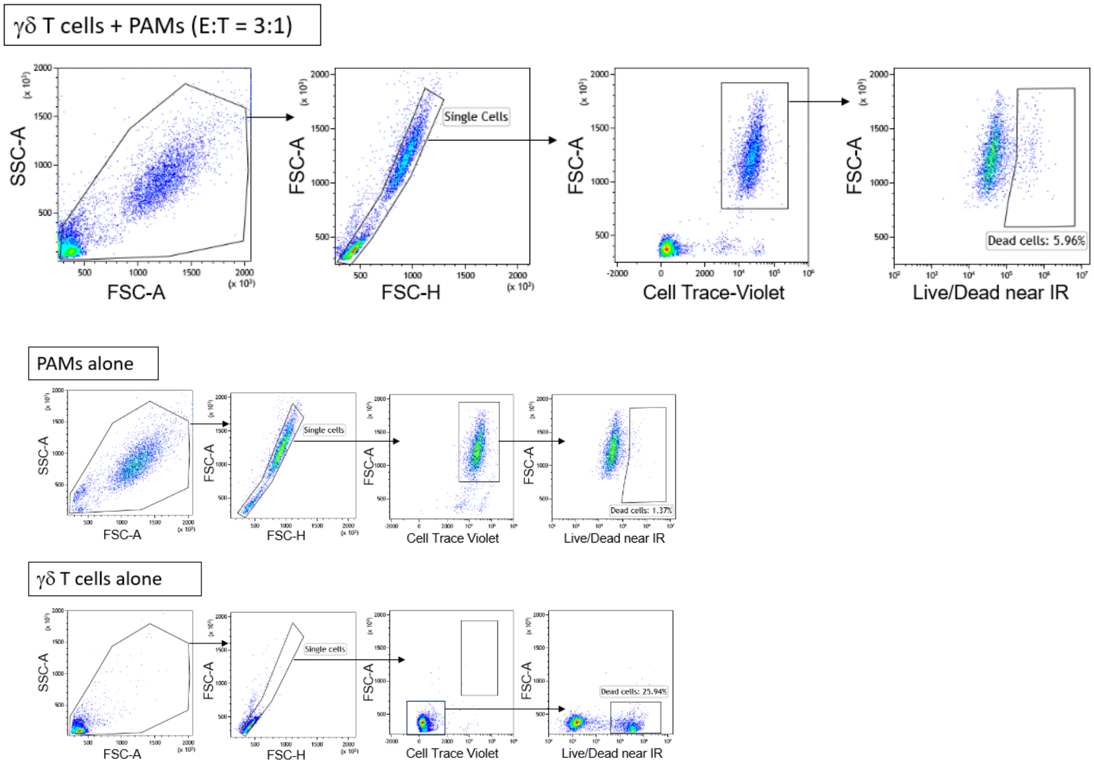

Supplementary Fig. 2. Schematic representation of the gating strategy for the analysis of PAM lysis mediated by γδ T cells. After the exclusion of doublets, PAMs were identified by Cell Trace Violet staining and size (FSC-A), followed by a viability assessment.

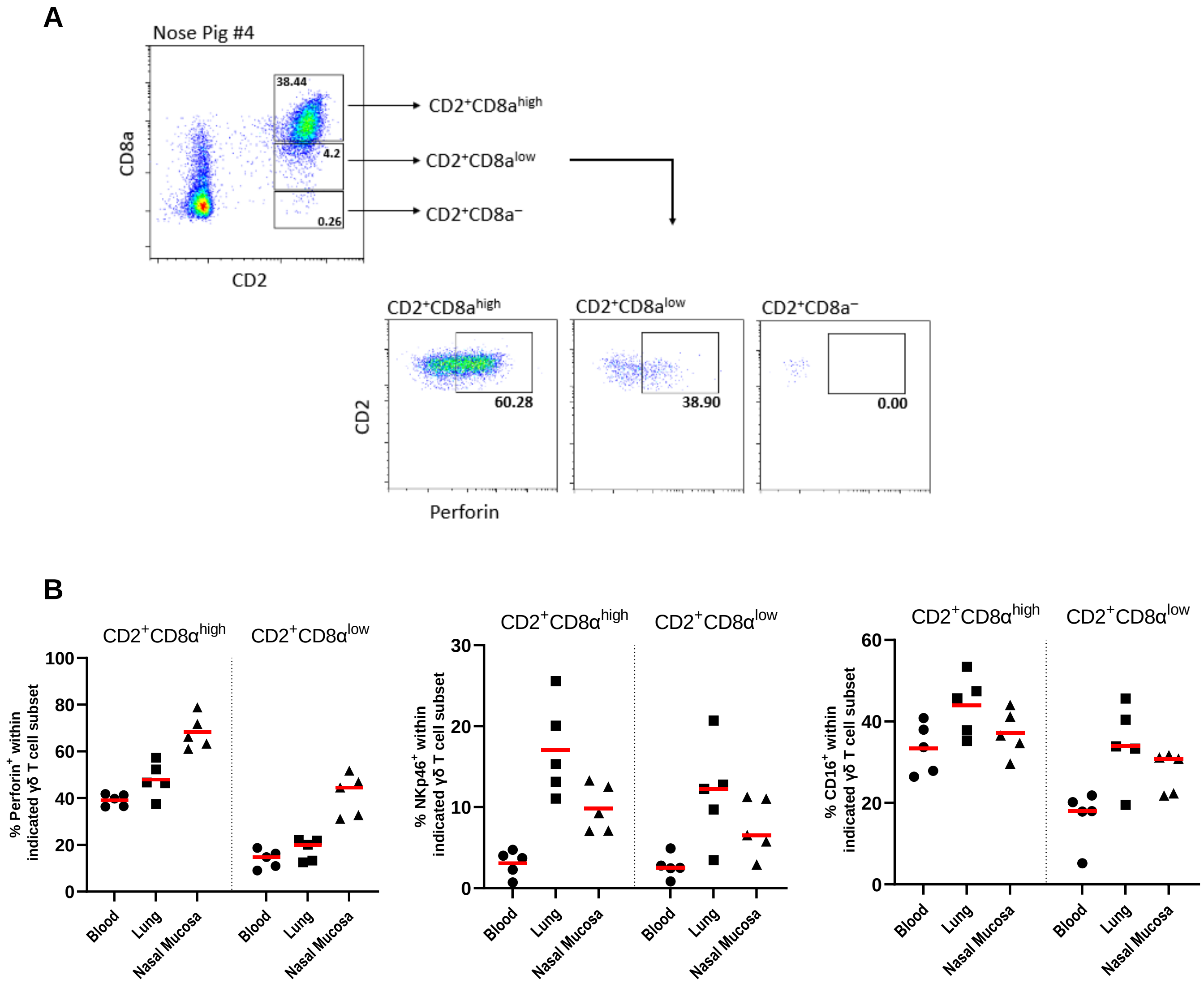

Supplementary Fig. 3. Cytotoxic marker (NKp46, perforin, CD16) expression in porcine CD2+ γδ T cells. After doublet and dead cell exclusion as shown in Supplementary Fig.1, γδ T cells were gated based on their expression of CD3 and δTCR, which was followed by an analysis of CD2/CD8α expression and subsequent subset-specific expression of perforin, NKp46 and CD16 **(A).** **(B)** shows the expression of perforin, NKp46 and CD16 within γδ T cell subsets isolated from blood, lung tissue or nasal mucosa. Red bars indicate mean values, and the data were graphed in GraphPad Prism 9.5.0.

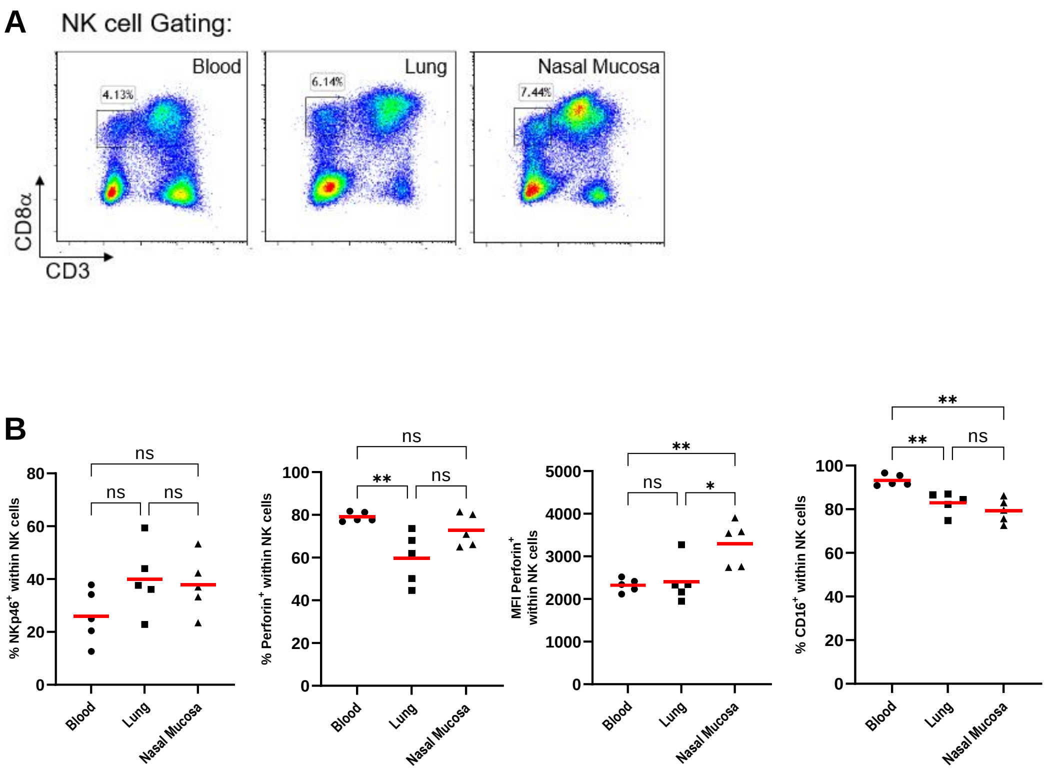

Supplementary Fig. 4. Cytotoxic marker (NKp46, perforin, CD16) expression in porcine NK cells. After doublet and dead cell exclusion as shown in Supplementary Fig.1, NK cells were gated based on their lack of CD3 and their expression of CD8α **(A). (B)** shows the expression of NKp46, perforin and CD16 within NK cells isolated from blood, lung tissue or nasal mucosa. Red bars indicate mean values, and the data were graphed in GraphPad Prism 9.5.0.

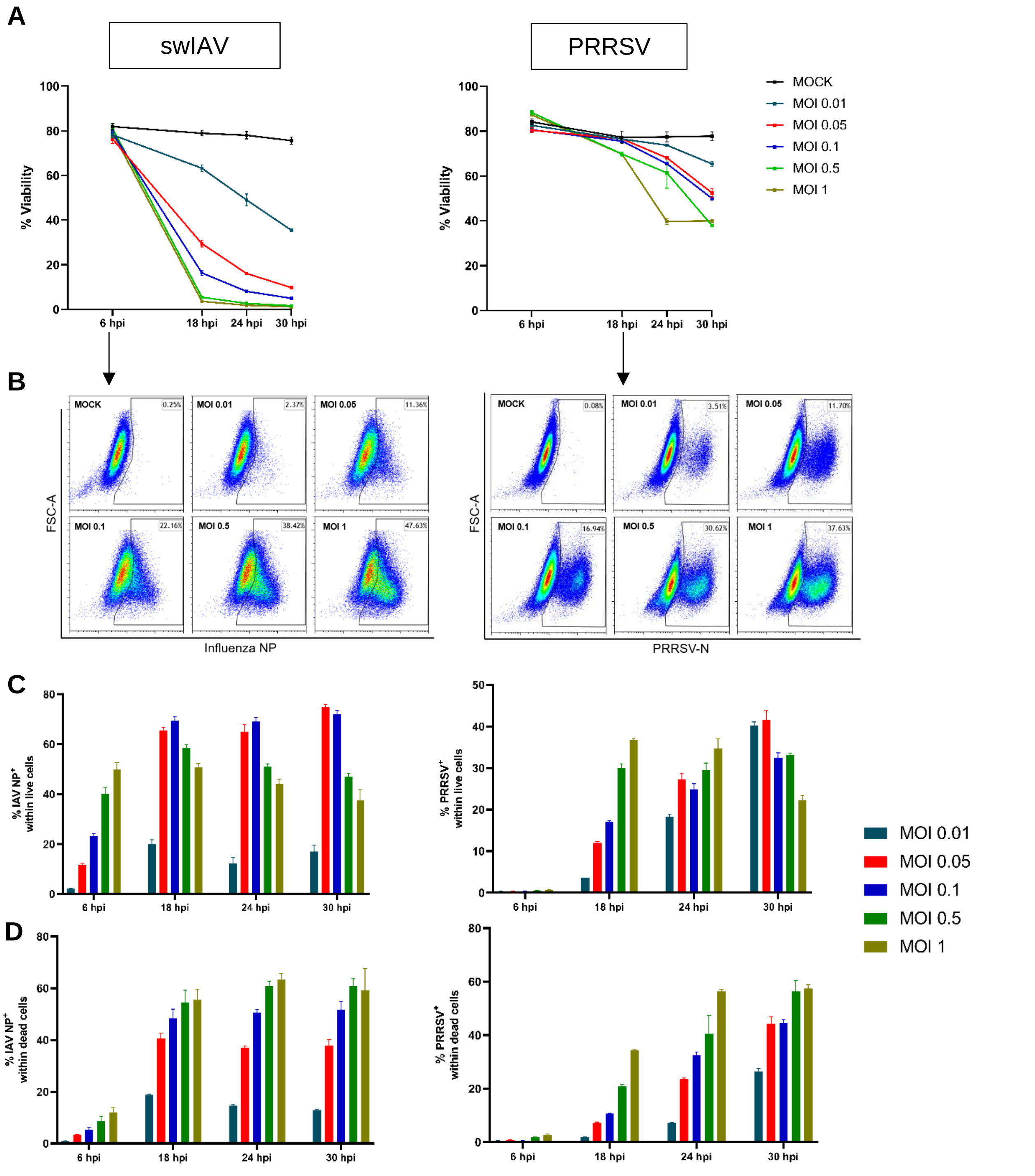

Supplementary Fig 5. SwIAV and PRRSV show different infection dynamics in PAMs. PAMs were mock-infected or infected with swIAV or PRRSV for 2 hours at varying MOIs and infection rate was evaluated at multiple timepoints as indicated. **(A)** shows the viability of cultured cells over time. Viability was assessed using flow cytometry and the LIVE/DEAD^{TM} Fixable Near-IR Dead Cell Stain Kit. **(B)** Representative plots

showing the frequency of intracellular viral protein within live cells at 6 hpi for swIAV and 18 hpi for PRRSV. The frequency of cells positive for intracellular viral protein for all time points and varying MOIs is shown in **(C)** and **(D),** with prior gating on live cells **(C)** or dead cells **(D)**. Each combination of MOI and time point was measured in triplicate, with SD being shown.

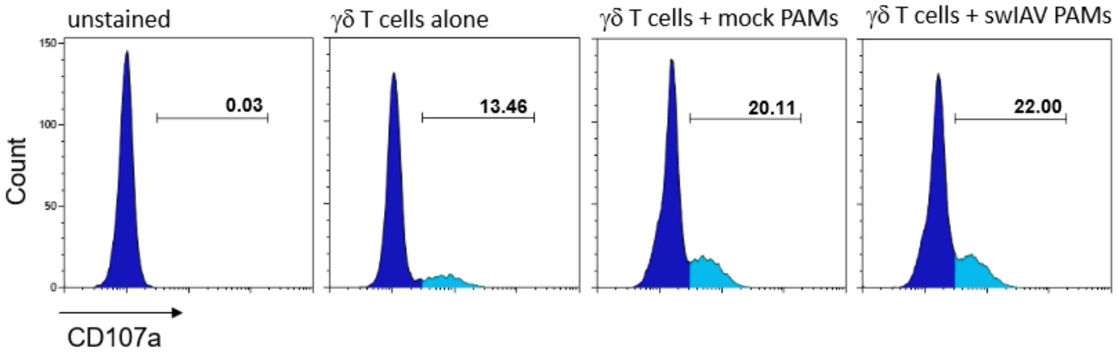

Supplementary Fig. 6. Schematic representation of the gating strategy for the analysis of degranulation of γδ T cells (CD107a expression). Purified and rested γδ T cells were co-cultured with CellTraceViolet-labeled PAMs (virus-exposed or mock) or cultured alone. A gate was established around singlets before drawing a gate surrounding intact cells. After this doublet and dead cell exclusion, γδ T cells were identified by the lack of CellTraceViolet Staining (as shown in Supplementary Fig.2). Gamma-delta T cells were then further analyzed for the expression of CD107a, as shown for one representative animal above.

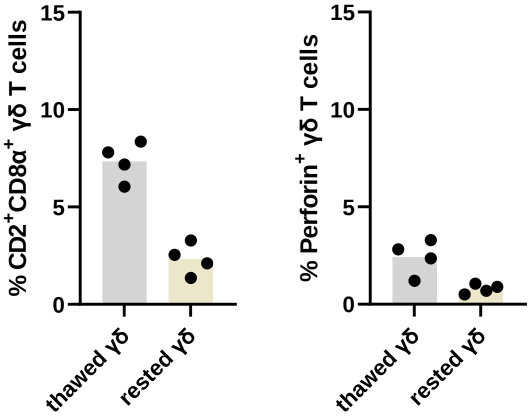

Supplementary Fig. 7. Perforin expression and CD2^{+}CD8α^{+} frequency within *ex vivo* or rested γδ T cells. Gamma-delta T cells were positively isolated (MACS) and then rested for 48h. After the resting period but before the co-culture with PAMs, they were harvested and stained for CD2, CD8α and perforin. For the *ex vivo* staining, PBMCs were thawed, and γδ T cells from matching animals were directly stained for perforin, CD2 and CD8α. After doublet and dead cell exclusion, the percentage of γδ T cells positive for perforin and frequency of CD2^{+}CD8α^{+} γδ T cells within total γδ T cells were analyzed.

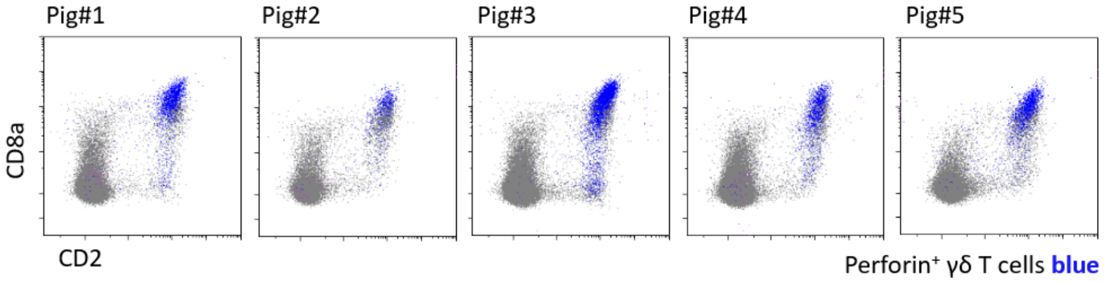

Supplementary Fig.8. Phenotype of perforin-expressing γδ T cells after 4h co-culture with mock-treated PAMs. Purified and rested γδ T cells were co-cultured with CellTraceViolet- labeled PAMs (mock). A gate was established around singlets before drawing a gate surrounding intact cells. After this doublet and dead cell exclusion, γδ T cells were identified by the lack of CellTraceViolet Staining (as shown in Supplementary Fig.2). Gamma-delta T cells were then further analyzed for the expression of CD2/CD8α and perforin (blue).

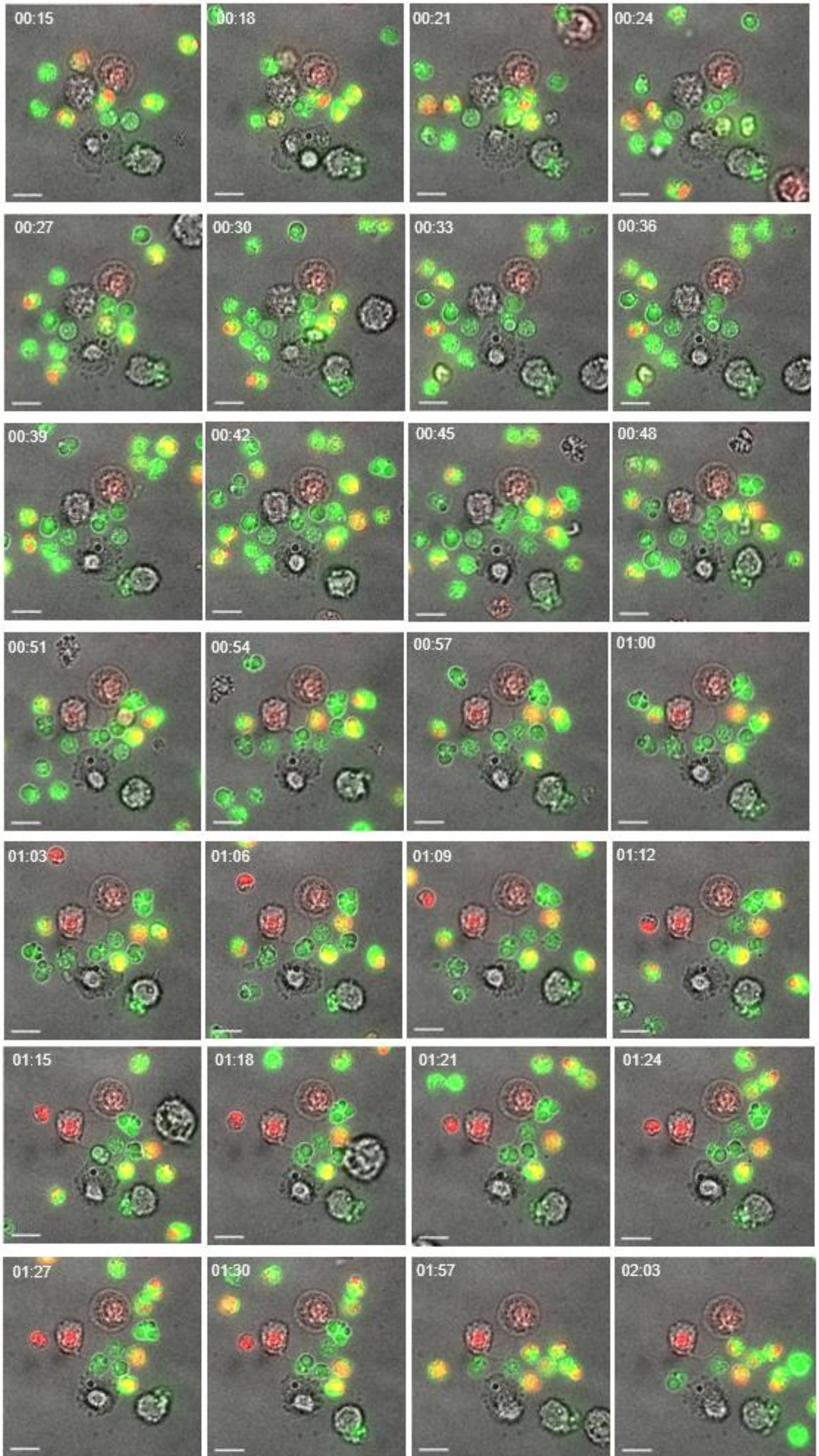

Supplementary Fig. 9. Time-lapse of $\gamma\delta$ T cell-PAM interaction in 3min intervals from 00:15 to 2:03 (hh:mm).

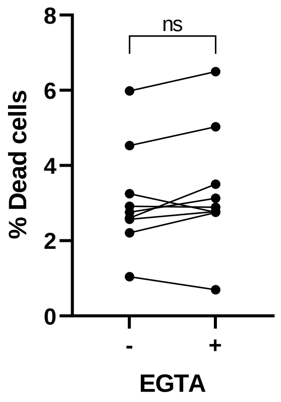

Supplementary Fig. 10. The presence of EGTA has no effect on the viability of PAMs cultured alone. PAMs were rested overnight, harvested, counted and replated in the presence or absence of EGTA (4 mM). After 4h, PAMs were harvested and viability was assessed by flow cytometry (Live/Dead^{TM} Fixable Near-IR Dead Cell Stain Kit)

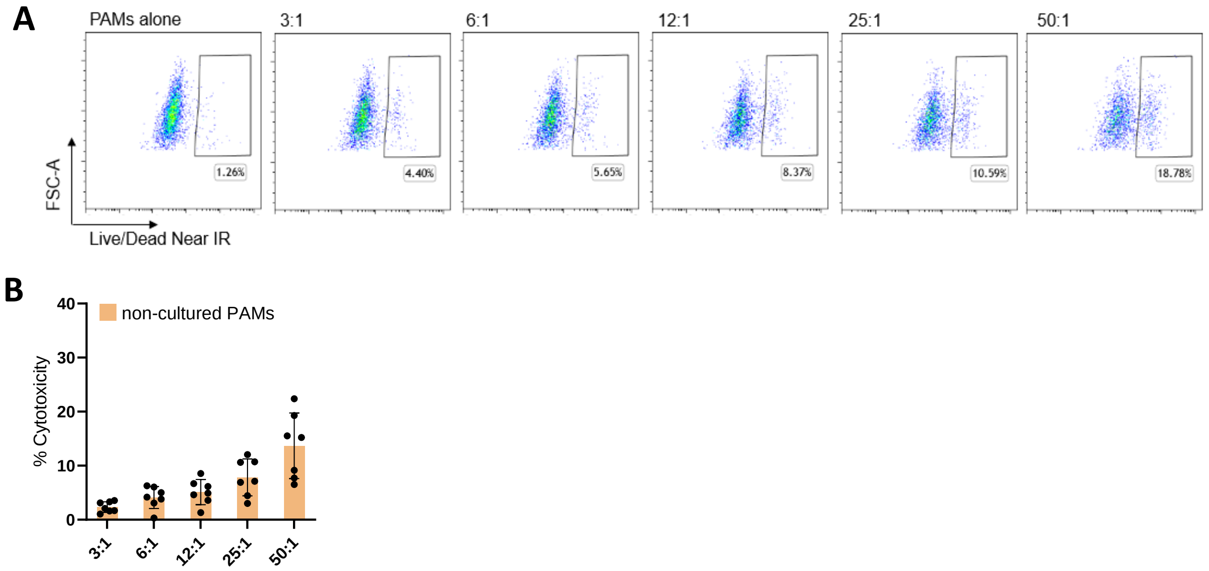

Supplementary Fig. 11. Porcine γδ T cells show lower cytotoxic activity against PAMs that were not cultured before the cytotoxicity assay. Gamma-delta T cells were cultured with previously cryopreserved PAMs. However, the PAMs were not cultured like the mock-treated and virus-exposed PAMs in Figure 2 prior to the cytotoxicity assay. **(A)** Representative flow plots showing the expression of viability dye in target cells (PAMs) without or with γδ T cells at varying target-to-effector ratios. The gating strategy is shown in Supplementary Figure 2. **(B)** Background-subtracted percentage of target cell death as measured by viability staining in non-cultured PAMs at different effector-to-target ratios. Background cell death for each experiment was calculated as the average of two wells of PAMs cultured without γδ T cells. Data are shown from n=7 individual 7-week-old pigs. Mean values and standard deviation (SD) are shown.

Supplementary Table 1. Primer Sequences and Efficiencies

Target	Sequence	Annealing temp. (°C)/ time (sec)	Product length	slope	Correlation Coefficient R^2	Primer Efficiency	Target sequence and reference
**RPL-19**	F: AACTCCCGTCAGCAGATCC<br>R: AGTACCCTTCCGCTTACCG	60/30	147	-3.298	0.999	101%	AF_435591; Bettin et al., 2023
**MIC2**	F: GAGCACTGAGACATGGCGTA<br>R: GAGGGAATGCAAGCCTCCTT	60/30	118	-3.522	0.997	92.3%	NM_00111427 4.1
**Fas**	F: CGTGAGGGTCAATTCTGCTGT<br>R: CTTGTCTGTGTAATCCTCCCCTTC	60/30	123	-3.483	0.999	93.8%	NM_213839.1 Lin et al., 2010
**DR5** TNFR10B -like	F: AACACCCTGAGCAATAGAGAC<br>R: CTCAAGCATTCTGTGGGGTC	60/30	200	-3.135	0.999	108.4%	XM_021073208 .1
**ICAM-1**	F: GGACCACAGAGACAAGTGGA<br>R: GCCACGACAAGTTAGCCAGT	60/30	161	-3.313	0.999	100.3%	NM_213816.1

MIC2: 1:4 serial dilution of cDNA pool (uninfected and PRRSV-infected PAMs)

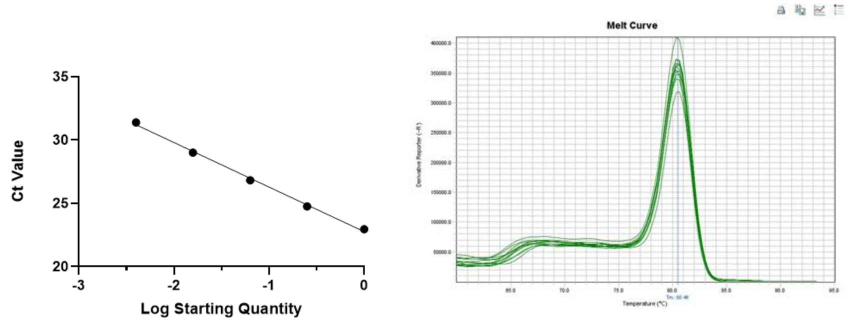

Fas: 1:4 serial dilution of cDNA pool (uninfected and PRRSV-infected PAMs)

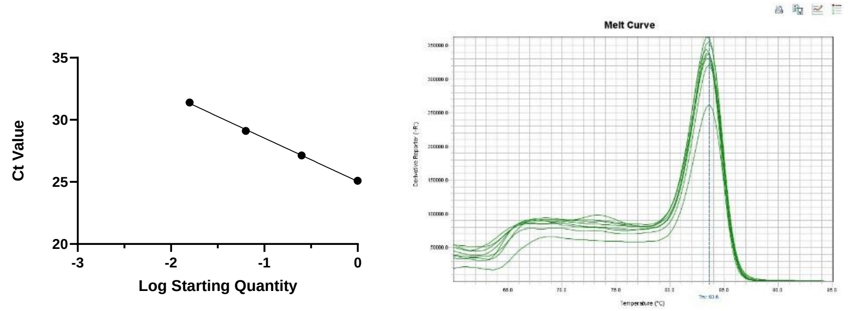

DR5: 1:2 serial dilution of cDNA pool (uninfected and PRRSV-infected PAMs)

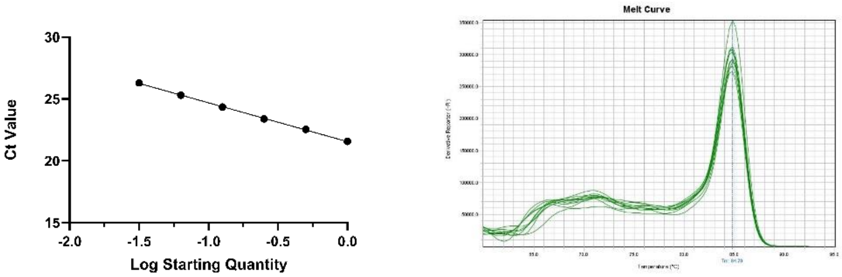

ICAM-1: 1:4 serial dilution of cDNA pool (uninfected and PRRSV-infected PAMs)

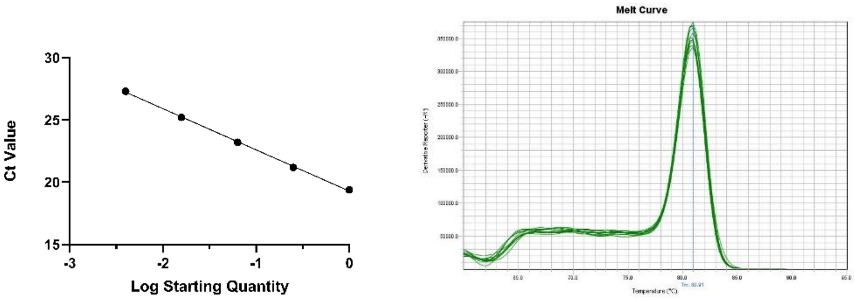

Supplement: Supplementary file 1 [file DataSheet_1.pdf]
